# Centromeric localization of αKNL2 and CENP-C proteins in plants depends on their centromere-targeting domain and DNA-binding regions

**DOI:** 10.1093/nar/gkae1242

**Published:** 2024-12-24

**Authors:** Surya Prakash Yalagapati, Ulkar Ahmadli, Aditya Sinha, Manikandan Kalidass, Siarhei Dabravolski, Sheng Zuo, Ramakrishna Yadala, Twan Rutten, Paul Talbert, Alexandre Berr, Inna Lermontova

**Affiliations:** Leibniz Institute of Plant Genetics and Crop Plant Research (IPK) OT Gatersleben, Corrensstr 3, 06466 Seeland, Germany; Leibniz Institute of Plant Genetics and Crop Plant Research (IPK) OT Gatersleben, Corrensstr 3, 06466 Seeland, Germany; Leibniz Institute of Plant Genetics and Crop Plant Research (IPK) OT Gatersleben, Corrensstr 3, 06466 Seeland, Germany; Leibniz Institute of Plant Genetics and Crop Plant Research (IPK) OT Gatersleben, Corrensstr 3, 06466 Seeland, Germany; Department of Biotechnology Engineering, Braude Academic College of Engineering, Snunit 51, P.O. Box 78, Karmiel 2161002, Israel; Anhui Provincial Key Laboratory of Molecular Enzymology and Mechanism of Major Metabolic Diseases, College of Life Sciences, Anhui Normal University, South Jiuhua Road 189, Wuhu 241000, China; Leibniz Institute of Plant Genetics and Crop Plant Research (IPK) OT Gatersleben, Corrensstr 3, 06466 Seeland, Germany; Leibniz Institute of Plant Genetics and Crop Plant Research (IPK) OT Gatersleben, Corrensstr 3, 06466 Seeland, Germany; Basic Sciences Division, Fred Hutchinson Cancer Research Center, 1100 Fairview Ave. N, Seattle, Wahington 98109, USA; Institut de Biologie Moléculaire des Plantes (IBMP), Centre National de la Recherche Scientifique (CNRS), UPR 2357, Université de Strasbourg, 12 rue du Général Zimmer, 67000 Strasbourg, France; Leibniz Institute of Plant Genetics and Crop Plant Research (IPK) OT Gatersleben, Corrensstr 3, 06466 Seeland, Germany

## Abstract

In eukaryotes, accurate chromosome segregation during cell division relies on the centromeric histone H3 variant, CENH3. Our previous work identified KINETOCHORE NULL2 (αKNL2) as a plant CENH3 assembly factor, which contains a centromere-targeting motif, CENPC-k, analogous to the CENPC motif found in CENP-C. We also demonstrated that αKNL2 can bind DNA *in vitro* in a sequence-independent manner, without the involvement of its CENPC-k motif. In this study, we show that the CENPC-k and CENPC motifs alone are insufficient for centromere targeting in *Nicotiana benthamiana* and *Arabidopsis thaliana*. *In silico* analysis identified adjacent DNA-binding regions near the CENPC-k and CENPC motifs, suggesting their role in centromeric DNA interaction. We further demonstrated that protein fragments containing these motifs effectively target centromeres. Deletion of these DNA-binding domains reduced the centromeric localization of αKNL2-C, while fusing CENPC-k to the non-specific DNA-binding domain of histone-like nucleoid structuring protein from *Escherichia coli* successfully targeted it to centromeres. Our findings suggest that the centromeric targeting of αKNL2 and CENP-C proteins relies on the CENPC-k/CENPC motifs, and that their sequence-independent DNA-binding activity enhances their centromere anchoring. These insights into the mechanisms of αKNL2 and CENP-C targeting may facilitate the engineering of kinetochore structures by directing chromatin-modifying proteins to centromeres.

## Graphical Abstract

**Graphical Abstract:**
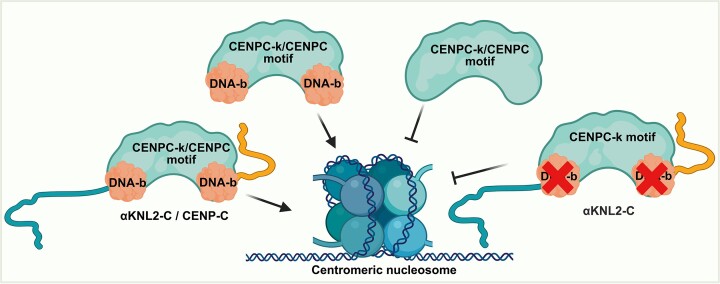

## Introduction

Centromeres are specific chromosomal positions where the kinetochore complex is established for the proper attachment of the spindle microtubules and the correct separation of chromosomes during mitosis and meiosis. Centromeric DNA sequences are highly variable even among closely related species and are composed of centromeric repeats and transposable elements (1,2). These sequences are neither required nor sufficient for centromere formation as so-called neocentromeres can be formed at atypical chromosomal regions such as chromosome arms or telomeres [reviewed by Scott and Sullivan (3)]. In centromeric chromatin, a significant proportion of nucleosomes is characterized by the presence of the centromere-specific histone H3 variant CENH3 instead of canonical H3. The presence of CENH3 homologs in all animals, fungi and plants studied so far has resulted in the assumption that CENH3-containing chromatin is a basic requirement for centromere function (4,5). However, Drinnenberg *et al.* (6) have demonstrated that several lineages of holocentric insects contain a CENH3-independent centromere. The loading of CENH3 into centromeric nucleosomes is a tightly regulated process that can be divided into three steps: licensing of the centromere, loading of CENH3 by histone chaperones and stabilization of the newly incorporated CENH3 in centromeric nucleosomes (7). The role of centromere-licensing factors was proposed for the Mis18 complex containing Mis18α, Mis18β and M18BP1/KINETOCHORE NULL2 (KNL2) proteins (8). Of this complex, only Mis18 proteins [Mis16, Mis19 (Eic1) and Mis20 (Eic2)] were found in yeast (9), and only M18BP1/KNL2—in *Caenorhabditis elegans* (10) and plants (7). Reduced *KNL2* expression in plants results in reduced level of CENH3 at the centromeres of meristematic nuclei, anaphase bridges during mitosis, micronuclei in pollen tetrads followed by a reduced growth rate and fertility (7).

Recently it was shown that in plants the *KNL2* gene underwent three independent ancient duplications, namely in ferns, grasses, and eudicots (11). Additionally, previously unclassified *KNL2* genes could be divided into the clades αKNL2 and βKNL2 in eudicots and γKNL2 and δKNL2 in grasses, respectively. Thus, in this study, *Arabidopsis* KNL2, which was previously reported, will be referred to as αKNL2 (7). All KNL2 homologs identified so far are characterized by the conserved N-terminal SANTA (SANT associated) domain. In vertebrates, the SANTA domain is present in parallel with the putative DNA-binding domain SANT. In contrast, in both KNL2 variants of plants, the SANT domain cannot be detected (7,11), although the C-terminal part of αKNL2 demonstrated DNA-binding capacity (12).

A conserved CENPC-k motif identified in α- and γKNL2 of plants and in KNL2 of non-mammalian vertebrates (12,13) was not found in β- and δKNL2 variants of plants and in mammals (11). The CENPC motif was originally considered as a typical feature of the CENP-C protein required for its targeting to the centromere and interaction with the CENH3 nucleosome (14). Later, the CENPC-k motif was shown to be similarly required for targeting αKNL2 protein homologs to centromeres in *Arabidopsis* (12), chicken (15) and frog (16). In addition, a recent study by Jiang *et al.* (17) showed that the CENPC-k motif of chicken KNL2 is able to bind directly to the CENH3 in the centromeric nucleosome. For new CENH3 deposition in human cells, M18BP1/KNL2, which lacks the CENPC-k motif (13), localizes to centromeres via CENP-C binding (18,19). In contrast, in *Xenopus* egg extract or chicken DT40 cells, the association of KNL2 containing the CENPC-k motif (13) with interphase centromeres does not require CENP-C (20,21). Despite the low sequence similarity between KNL2 and CENP-C, the two proteins share the presence of a CENPC-like motif and the ability to bind DNA and RNA molecules. Both αKNL2 and CENP-C proteins of plants can bind DNA in a sequence-independent manner *in vitro* while *in vivo*, they are preferentially associated with the centromeric repeats (12,22). Additionally, human CENP-C has shown interaction with DNA in a similar way (23). The fact that the C-terminal fragment of αKNL2 can target centromeres of *Nicotiana benthamiana*, even though its centromeric sequences differ from those of *Arabidopsis thaliana*, further confirms that the targeting of αKNL2 to centromeres and its binding to DNA do not depend on the sequence of centromeric DNA (12).

Here we show that the expression of fusion constructs of CENPC-k and CENPC motifs of αKNL2 and CENP-C proteins, respectively, with enhanced yellow fluorescent protein (EYFP) resulted in a distribution of fluorescence in the nucleoplasm and cytoplasm of transiently transformed *N. benthamiana* and stably transformed *A. thaliana*, demonstrating that these motifs alone are insufficient for targeting centromeres. We identified putative DNA-binding regions near the CENPC motifs of αKNL2 and CENP-C proteins and demonstrated that protein fragments containing CENPC motifs and DNA-binding sites can target centromeres. Moreover, deleting one of the DNA-binding sites from the C-terminus of αKNL2 showed a reduced localization to centromeres, whereas the deletion of both abolished its centromeric localization. Using *in silico* analysis and electrophoretic mobility shift assay (EMSA), we have also demonstrated that the deletion of putative DNA-binding regions reduces the interaction of αKNL2-C with the centromeric repeat *pAL1*. Targeting protein fragments containing the CENPC-k motif in combination with bacterial DNA-binding region(s) to centromeres showed that any DNA-binding regions with sequence-independent DNA-binding ability are sufficient to anchor kinetochore proteins to centromeres.

## Materials and methods

### Plasmid construction and site-directed mutagenesis

All fragments including the CENPC-k and CENPC motifs with and without DNA-binding regions (Supplementary Tables S1–S3) were amplified from the αKNL2 and CENP-C cDNA clones in the pDONR221 (Invitrogen) vector using primers listed in Supplementary Table S4. Subsequently, all fragments were separately cloned into the pDONR221 vector via the Gateway Cloning (BP reaction). To delete the sequences between the DNA-binding regions and the CENPC-k motif (amino acids 477–538 and 573–580), the CENPC-k_DNA-binding/pDONR221 construct was subjected to PCR mutagenesis using the Phusion Site-Directed Mutagenesis Kit (Thermo Fisher Scientific) with the primer pairs listed in Supplementary Table S4. Deletion constructs for the upstream DNA-binding region (amino acids 468–477) and the downstream DNA-binding region (amino acids 581–598) of the CENPC-k motif of αKNL2-C were generated using the αKNL2-C/pDONR221 construct, with the corresponding primer pairs as indicated in Supplementary Table S4. All plasmid clones were prepared for sequence analysis using the QIAprep kit according to the instructions of the supplier (QIAGEN). From pDONR221 clones, the open reading frames were recombined via Gateway LR reaction (Invitrogen) into the two attR recombination sites of the Gateway-compatible vectors pGWB641 and pGWB642 (https://shimane-u.org/nakagawa/) to study the localization of proteins *in vivo*.

To assess the influence of a non-plant DNA-binding domain on the centromeric localization of the CENPC-k motif, the non-specific DNA-binding domain (Nsdbd) was isolated from the bacterial histone-like nucleoid structuring protein (H-NS). Firstly, the genomic DNA from *Escherichia coli* was purified following the Wizard Genomic DNA Purification Kit (Promega) protocol and served as a template for PCR amplification. The Nsdbd was then amplified from this template using primers listed in Supplementary Table S4. Following gel purification, the PCR product was ligated into the pGEM-T Easy vector (Promega). Subsequently, after validation through sequencing, the Nsdbd fusion was re-amplified with Gateway-compatible primers (Supplementary Table S4) and cloned into the pDONR221 vector (Invitrogen). Finally, the Nsdbd fragment was recombined from a pDONR221 clone through the Gateway LR reaction into the Gateway-compatible vector pB7WGF2 (24). To generate fusion constructs of CENPC-k motif with Nsdbd DNA fragments of ATG-Nsdbd-CENPC-k-Nsdbd and ATG-Nsdbd-CENPC-k (Supplementary Table S3), they were synthesized by BioCat company (https://www.biocat.com/) and provided as clones in pDONR221 vectors. From pDONR221 clones, the open reading frames were recombined via the Gateway LR reaction (Invitrogen) into the two attR recombination sites of the Gateway-compatible vectors pGWB641 and pGWB642.

### Plant transformation and cultivation

*Agrobacterium tumefaciens*-mediated transient transformation of *N. benthamiana* plants was performed according to (25). For each construct, transient transformation of *N. benthamiana* was repeated at least three times.

Plants of *Arabidopsis* accession Columbia-0 were transformed according to the floral dip method (26). T1 transformants with pGWB641 and pGWB642 vector were selected on Murashige and Skoog (MS) medium (27) containing 20 mg/l of phosphinotricine. Growth conditions in a cultivation room were 21°C, 8 h light/18°C, 16 h dark or 21°C, 16 h light/18°C, 8 h dark.

### Nuclei isolation and immunostaining

To analyze the colocalization of EYFP-fused protein fragments with *N. benthamiana* CENH3 and to quantify the number of nuclei with centromeric signals, nuclei were isolated from *N. benthamiana* leaves following the protocol described by Doležel *et al.* (28). Isolated nuclei were then immunostained with *N. benthamiana* CENH3 antibodies. This preparation method preserved the *in vivo* EYFP fluorescence of the expressed proteins, allowing the detection of EYFP signals even after immunostaining.

Immunostaining of nuclei/chromosomes was performed according to (29). *N. benthamiana* CENH3 protein was detected with rabbit polyclonal antisera (1:2000; LifeTein, https://www.lifetein.com) against the N-terminal peptide of CENH3 and with goat anti-rabbit rhodamine (1:200; Jackson Immuno Research Laboratories, https://www.jacksonimmuno.com). EYFP fusion protein variants in *A. thaliana* were detected with rat monoclonal antisera (1:2000; Chromotek, https://www.ptglab.com/) against GFP, which also recognizes EYFP protein and with goat anti-rat Alexa 488 (1:200; Jackson Immuno Research Laboratories, https://www.jacksonimmuno.com). *A. thaliana* CENH3 protein was detected with rabbit polyclonal antisera (1:2000; LifeTein, https://www.lifetein.com) against the N-terminal peptide of CENH3 (30) along with goat anti-rabbit rhodamine (1:200; Jackson Immuno Research Laboratories, https://www.jacksonimmuno.com).

### Microscopy analysis of fluorescent signals

*In vivo* fluorescence analysis was conducted on the epidermal cell layers of *N. benthamiana* leaves two days post-infiltration and on the root tips of 7-day-old seedlings of stably transformed *A. thaliana*. Fluorescence was detected with a confocal LSM 780 laser scanning microscope (Carl Zeiss, Jena, Germany) using a 40×/1.2 water immersion objective. EYFP was excited with a 488-nm laser line and fluorescence was analyzed with a 505–550 nm band-pass. Images were recorded with the LSM software Zen Black release 3.2.

### *In silico* prediction of the DNA-binding ability of proteins

To predict the DNA-binding ability of M18BP1/KNL2 proteins of plants, mammalian and non-mammalian vertebrates, and *C. elegans*, structural models were constructed using the SWISS‐MODEL (31) for Q7XVZ3, B8A9Q3, A0A8M2B5N0, A0A8V0Z4U5 or down-loaded from the AlphaFold Protein Structure Database (https://alphafold.ebi.ac.uk/) (32,33) for other proteins. The presence of DNA-binding regions within selected proteins was detected with the following sequence- and structure-based on-line tools: DNABIND (34), DRNApred (35), DNAgenie (36) and NucBind (37).

### Homology modelling and protein-DNA interaction by molecular docking

The protein sequence of the centromeric protein αKNL2 was retrieved from the UniProtKB database (https://www.uniprot.org/) in FASTA format. Homology modeling of the αKNL2-C and CENP-C proteins using the FASTA sequence was performed using I-TASSER (Iterative Threading ASSEmbly Refinement). I-TASSER is a hierarchical approach to predicting protein structure and structure-based function annotation. The program detects structural templates from the PDB (Protein Data Bank) using a multiple threading method (LOMETS), then builds full-length atomic models using iterative template-based fragment assembly simulations. I-TASSER is available at https://zhanggroup.org/I-TASSER/.

HDOCK is an online server used to identify protein–protein and protein-DNA/RNA docking based on a hybrid algorithm of template-based modeling and *ab initio* free docking (http://hdock.phys.hust.edu.cn/). For the centromeric DNA sequence, the *pAL1* repeat sequence has been retrieved. Modeled PDB structures were submitted to the HDOCK server for the protein-DNA interaction. The interaction between two molecules is determined based on two scores, as the docking score and the confidence score. A more negative docking score, such as −200 or lower, indicates a stronger binding model. In accordance with the confidence score, the two molecules would be very likely to bind if the score is above 0.7, the two molecules would be possible to bind when the score is between 0.5 and 0.7, and if it is below 0.5, the two molecules would be unlikely to bind.

### Electrophoretic mobility shift assay

The pF3A WG vectors (Promega) containing the coding sequences of αKNL2-C, αKNL2-CΔDNAb (1), αKNL2-CΔDNAb (2) and αKNL2-CΔDNAb(1,2) fused with a FLAG tag were used for protein expression. Expression was carried out in the TNT SP6 High Yield Wheat Germ system (Promega) following the manufacturer’s protocol, with the reaction incubated for 2 h at 25°C. Protein expression was validated by Western blot analysis using an anti-FLAG antibody (Sigma). For the EMSA assays, the *Arabidopsis* centromeric repeat *pAL1* was amplified by PCR using fluoroscently labeled (IR-DYE-700) primers. The probe was purified using an oligonucleotide purification kit (BioRad). The binding reaction was set up using the Odyssey EMSA kit (LI-COR). Wheat germ-expressed proteins (3 μl) were incubated with 1 ng of the probe for 30 min at room temperature. Following incubation, 2 μl of orange loading dye was added to the reaction and the samples were loaded and separated on a 5% native polyacrylamide gel at 4°C with 70 V until the dye front reached the bottom of the gel. Gels were imaged using the LI-COR Odyssey scanner.

### Quantification and statistical analysis

Fluorescent signals in *N. benthamiana* were analyzed for each fusion construct across 150 nuclei (3 samples of 50 nuclei each). Quantification was performed by assessing the co-localization of EYFP signals with *N. benthamiana* CENH3 at centromeres. Error bars represent the mean ± SEM from a minimum of three independent experiments or samples.

## Results

### CENPC-k and CENPC motifs are necessary but insufficient for independent centromere targeting

**Figure 1.**
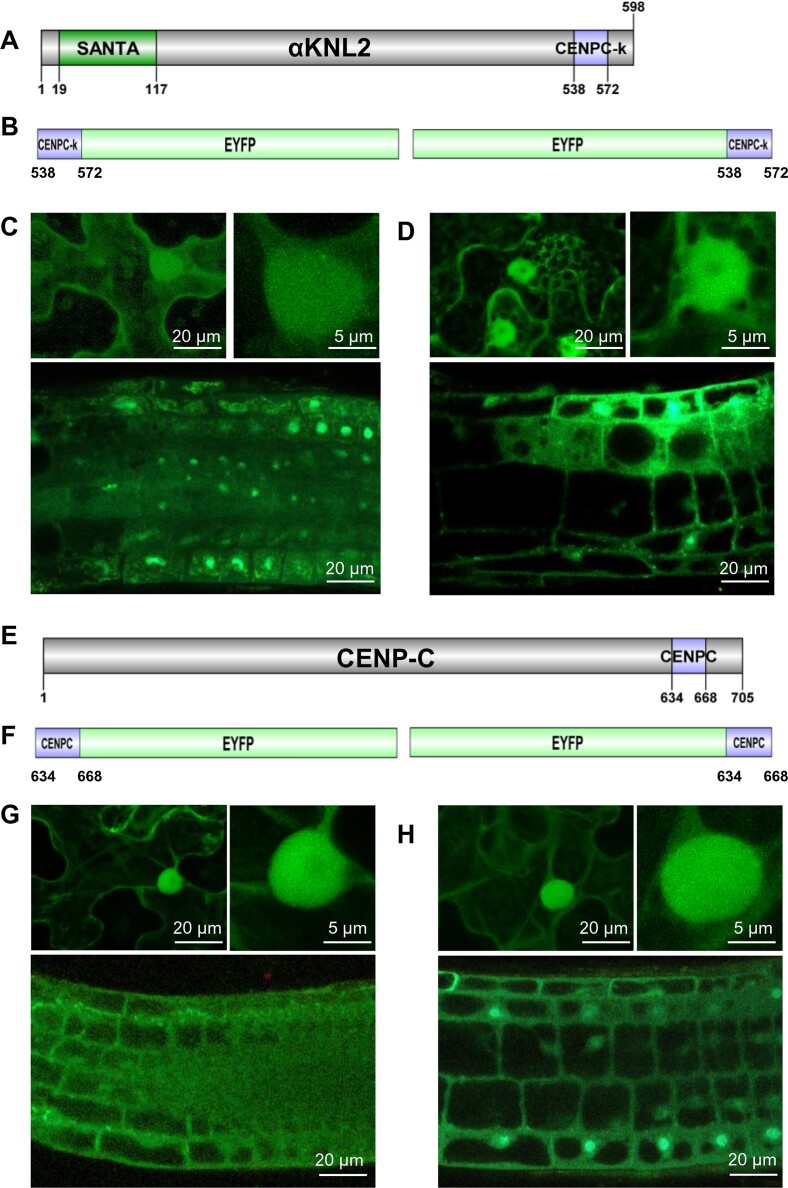
The CENPC-k and CENPC motifs of the αKNL2 and CENP-C proteins are unable to target centromeres independently in *N. benthamiana* and *A. thaliana*. (**A**) Schematic representation of the αKNL2 protein, showing the conserved SANTA domain and CENPC-k motif. Numbers indicate amino acid positions. (**B**) CENPC-k-EYFP and EYFP-CENPC-k fusion constructs. (**C**and **D**) Subcellular localization of the corresponding fusion proteins in leaves of transiently transformed *N. benthamiana* (upper panels) and roots of stably transformed *A. thaliana* (lower panels). In all cases, fluorescent signals were detected in the nucleoplasm and cytoplasm. No signals corresponding to centromeres were observed. (**E**) Schematic representation of the CENP-C protein with the conserved CENPC motif. Numbers indicate amino acid positions. (**F**) CENPC-EYFP and EYFP-CENPC fusion constructs. (**G**and**H**) Subcellular localization of the corresponding fusion proteins in transiently transformed *N. benthamiana* (upper panels) and stably transformed *A. thaliana*. (lower panels). In all cases, fluorescent signals were detected in the nucleoplasm and cytoplasm. No signals corresponding to centromeres were observed.

The CENPC-k motif is essential for targeting the αKNL2 protein to centromeres in *Arabidopsis*. The mutagenesis of conserved amino acids or complete deletion of the CENPC-k motif abolished the centromeric localization of αKNL2. However, this centromeric localization can be restored by replacing the CENPC-k motif of αKNL2 with the motif from the CENP-C protein (Sandmann *et al.* 2017). The CENPC-k motif (538–572 aa) is located at the C-terminal part of the αKNL2 protein (Figure 1A). To determine whether a CENPC-k motif alone is sufficient for centromeric targeting, it was fused to an EYFP tag driven by the 35S promoter. Since the fusion orientation can strongly influence the subcellular localization of the protein, constructs with EYFP fused either to the N- or C-terminus of the CENPC-k motif were designed (Figure 1B). To analyze the subcellular localization of EYFP-tagged CENPC-k motifs in plants, young *N. benthamiana* leaves were transiently transformed using *Agrobacterium* suspensions carrying the respective constructs. Two days after transformation, fluorescent signals were observed in both the nucleoplasm and cytoplasm across all samples. The fluorescence was distributed homogeneously in the nucleoplasm, and no signals corresponding to centromeres were detected (Figure 1C and D, upper panels). To test the expression of these constructs in a homologous system and in various plant tissues and organs, stable transformants of *Arabidopsis* were generated, and T1 transgenic plants were obtained. Analysis of at least 10 independent transgenic lines for each construct revealed fluorescent signals predominantly in the cytoplasm and nucleoplasm of root tips, consistent with our observations in *N. benthamiana* (Figure 1C and D, lower panels).

To determine the centromere targeting efficiency of the CENPC motif (634–668 aa) from CENP-C, this motif was fused with EYFP in both orientations (Figure 1E and F). Similar to the CENPC-k motif, the localization analysis in *N. benthamiana* revealed that both fusion variants localized to the nucleoplasm and cytoplasm (Figure 1G and H, upper panels). Furthermore, stable *Arabidopsis* lines carrying these constructs did not show specific signals at centromeres and instead exhibited localization to the nucleoplasm and cytoplasm in root tips (Figure 1G and H, lower panels).

### *In silico* identification of DNA-binding regions in αKNL2 and CENP-C proteins

The results presented above clearly demonstrated that the CENPC-k and CENPC motifs alone are insufficient for targeting centromeres. Sandmann *et al.* (12) showed earlier that the C-terminal region of αKNL2, harboring the centromere-targeting CENPC-k motif, binds to centromeric DNA *in vitro* and preferentially associates with the centromeric DNA *in vivo*. Similarly, the CENP-C protein of maize has been shown to bind DNA (22). We thus hypothesized that incorporating additional putative DNA-binding region(s) adjacent to the CENPC-k or CENPC motifs might enhance centromere targeting.

**Figure 2.**
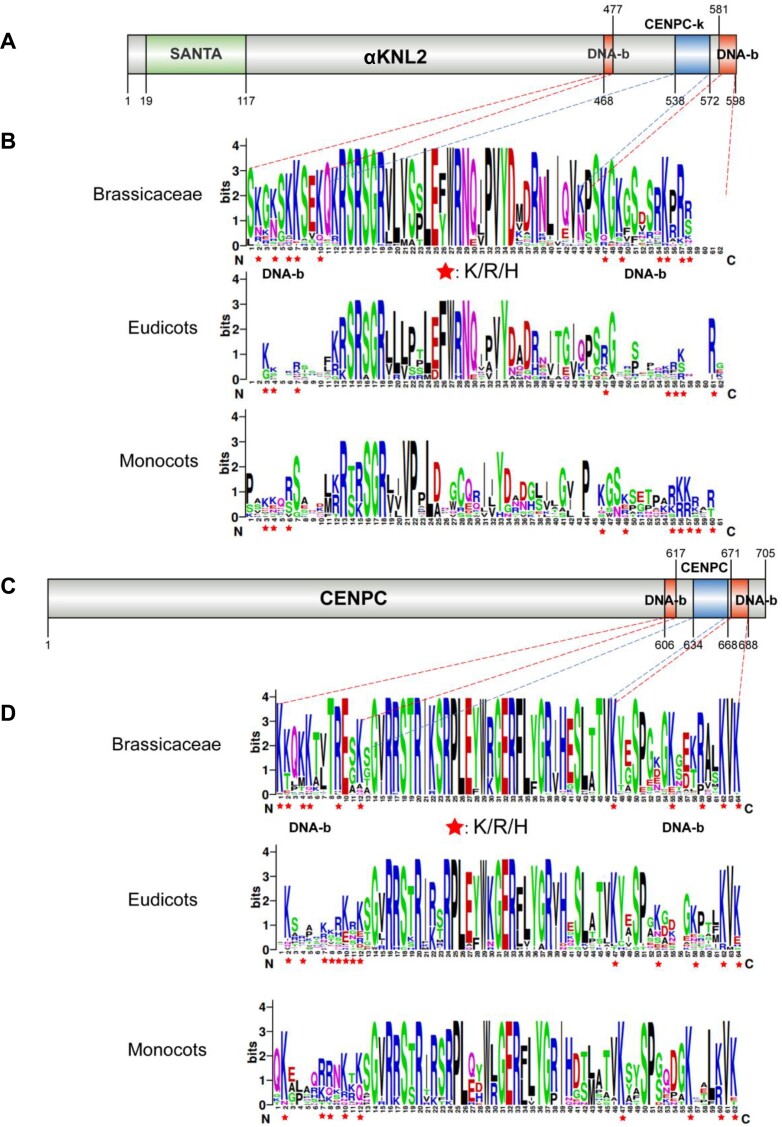
The DNA-binding regions of αKNL2 and CENP-C proteins are less conserved than the CENPC-k and CENPC motifs across different plant species. (**A and C**) Domain maps of αKNL2 and CENP-C proteins showing the positions of the SANTA domain, CENPC-k, CENPC motifs and DNA-binding regions. (**B**and**D**) Alignments of CENPC-k, CENPC motifs and DNA-binding regions presented in LOGO format for comparison of similarities and differences among the Brassicaceae family, Eudicot and Monocot plant species. Positively charged amino acid residues (R/K/H) in the DNA-binding regions from αKNL2 and CENP-C orthologs are marked by stars.

To predict putative DNA-binding regions within *Arabidopsis* αKNL2 and CENP-C proteins (Supplementary dataset 1), we employed a combination of sequence-based and structure-based predictors, including DP-Bind (38), DRNApred (35), DNAgenie (36) and GraphBind (39). All predicted data consistently identified DNA-binding sites within both αKNL2 and CENP-C proteins (Figure 2A and C, Supplementary Table S5), corroborating our previous findings. Specifically, DNA-binding regions within αKNL2 were predicted at amino acid positions 468–477 [αKNL2-C_DNAb(1)] and 581–598 [αKNL2-C_DNAb(2)] (Figure 2A), and at positions 606–617 [CENPC_DNAb(1)] and 671–688 [CENPC_DNAb(2)] in CENP-C (Figure 2C). These regions are located near the CENPC-k and CENPC motifs, respectively. In addition, putative DNA-binding sites were also identified within the conserved CENPC-k and CENPC motifs themselves. Since deletion of the CENPC-k motif from the C-terminal part of αKNL2 does not influence its ability to interact with DNA *in vitro* (12), these DNA-binding sites may not be essential, at least for αKNL2. To have a broad view of the conservation and variation of DNA-binding regions in αKNL2 and CENP-C, we produced an alignment of αKNL2 and CENP-C proteins across different plant species (Figure 2B and D). While numerous residues within these regions exhibited variability among homologs, the presence of conserved positively charged amino acid residues, responsible for DNA-binding, was observed in nearly all homologs. This implies that despite variations, αKNL2 and CENP-C retain their ability to bind DNA across plant species. To test whether this ability to bind DNA/RNA is a common feature of M18BP1/KNL2 proteins, we extended our *in silico* analysis and included βKNL2 from *A. thaliana*, δKNL2 from *Oryza sativa* and M18BP1 proteins from *Homo sapiens*, *Mus musculus*, *Danio rerio*, *Gallus gallus*, and *Caenorhabditis elegans*. Analysis using sequence- and structure-based online tools [DNABIND (34), DRNApred (35), DNAgenie (36) and NucBind (37)], showed that all selected proteins have the ability to bind nucleic acids (Table 1).

**Table 1.** *In silico* prediction of DNA-binding regions within KNL2 homologues

Protein ID	DNABIND	DRNApred	DNAgenie	NucBind
*A. thaliana -* αKNL2 UniProt: F4KCE9	+	+	+	+
*A. thaliana -* βKNL2 UniProt: Q8RWD7	+	-	+	+
*H. sapiens -* M18BP1 UniProt: Q6P0N0	+	+	+	+
*M. musculus* - Mis18-binding protein 1 UniProt: Q80WQ8	+	+	+	+
*D. rerio* - Mis18-binding protein 1 isoform X1 UniProt: A0A8M2B5N0	+	+	+	+
*G. gallus* - MIS18 binding protein 1 UniProt: A0A8V0Z4U5	+	+	+	+
*C. elegans* - Kinetochore null protein 2 UniProt: O44548	+	+	+	+
*O. sativa subsp. japonica* - γKNL2 UniProt: Q7XVZ3	+	-*	- **	+
*O. sativa subsp. indica* - δKNL2 UniProt: B8A9Q3	+	+	+	+

*only 2 DNA-binding aa, no RNA-binding aa

**only 2 DNA-binding aa, many RNA-binding aa

### Molecular docking analysis predicts interaction of αKNL2-C and CENP-C DNA-binding regions with centromeric DNA (*pAL1*)

**Figure 3.**
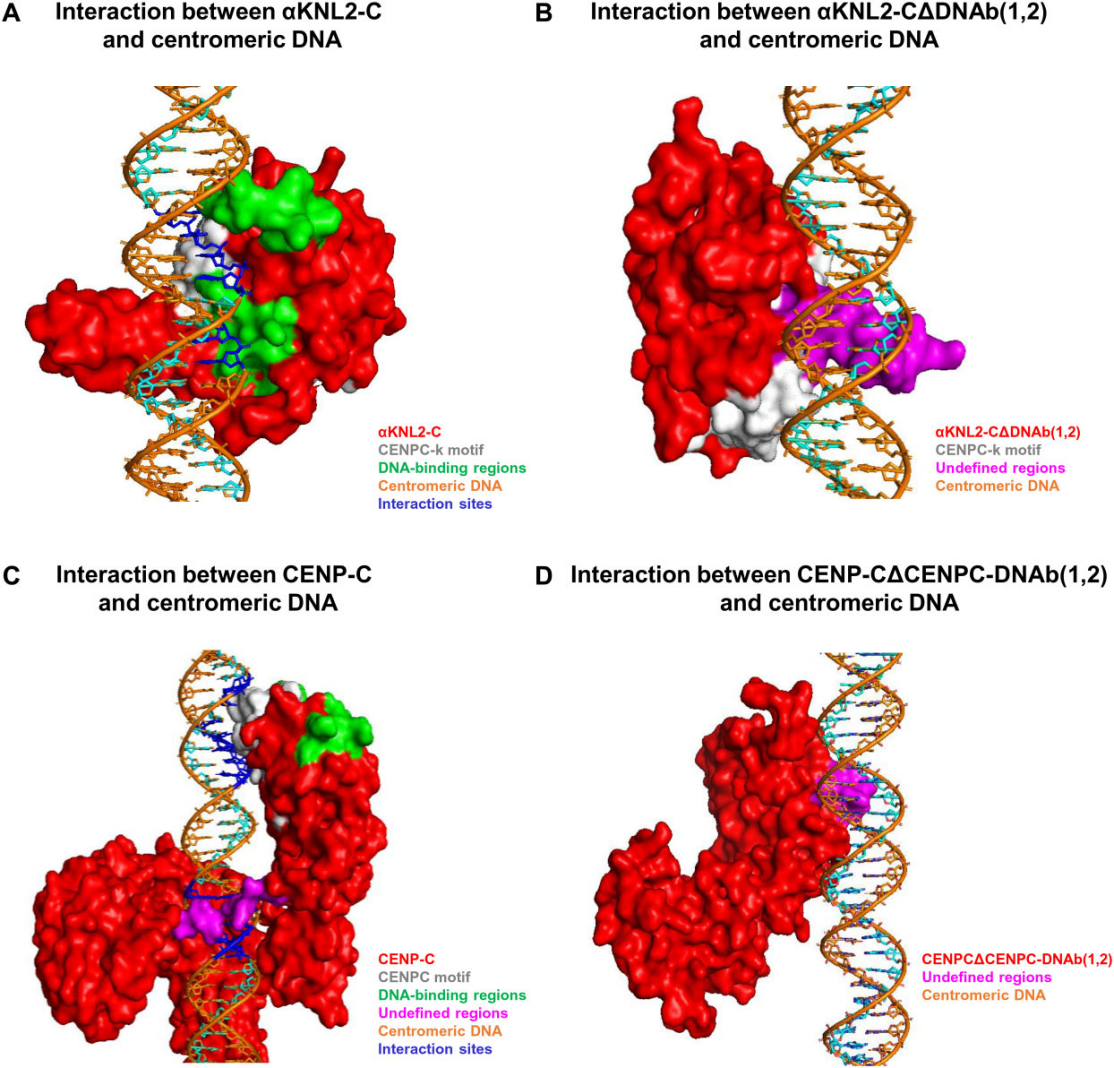
Interaction of αKNL2 and CENP-C proteins with centromeric DNA through molecular docking. (**A**) Docking of αKNL2-C with centromeric DNA (*pAL1*) demonstrates interactions mediated by DNA-binding regions and the CENPC-k motif. (**B**) Deletion of DNA-binding regions reduces the interaction between the αKNL2 and *pAL1*. However, the αKNL2-CΔDNAb(1,2) protein retained its association with centromeric DNA through the CENPC-k motif and additional undefined regions. (**C**) Docking of CENP-C with centromeric DNA reveals interactions facilitated by DNA-binding regions, the CENPC motif and other undefined regions. (**D**) Deletion of the CENPC motif and DNA-binding regions reduces the interaction between CENP-C and *pAL1* DNA. However, residual binding of CENP-CΔCENPC-DNAb(1,2) to *pAL1* DNA was observed via undefined regions. The protein is shown in surface rendering, and the DNA in cartoon stick models.

Following the identification of the putative DNA-binding sites on αKNL2 and CENP-C proteins, the interactions between their DNA-binding regions and centromeric DNA (*pAL1*) were investigated using molecular docking analyses. The three-dimensional structure of the αKNL2-C protein was modeled using I-TASSER, revealing a configuration comprising seven helices connected by four sheets via loops (Supplementary Figure S1A). To assess the interaction between αKNL2-C and *pAL1* DNA, the three-dimensional structure of αKNL2-C was docked with the *pAL1* DNA sequence using H-DOCK. This analysis revealed that the C-terminal part of αKNL2, which includes the DNA-binding regions, interacts with centromeric DNA. The top model showing the highest docking and confidence scores of −238.2 and 0.85, respectively, was selected for further analysis using PyMol. Our results show that both DNA-binding regions of αKNL2-C, as well as a portion of the CENPC-k motif, interact with centromeric DNA (Figure 3A). Subsequently, the αKNL2-C protein structure was modeled after deleting both DNA-binding regions [αKNL2-CΔDNAb(1,2)]. Docking this truncated version with centromeric DNA resulted in reduced docking and confidence scores to −179.1 and 0.53, respectively, with only partial interaction observed between the CENPC-k motif and DNA (Figure 3B, Supplementary Figure S1C). Thus, the DNA-binding regions near the CENPC-k motif may play a crucial role in mediating the interactions with DNA and anchoring αKNL2 to centromeres.

The docking analysis was also carried out for the I-TASSER-modelled CENP-C protein with centromeric DNA (*pAL1*). The modelled CENP-C protein exhibits 22 helices and 10 sheets interconnected by loops (Supplementary Figure S1B). Results from the top-predicted model revealed an interaction between the CENP-C protein and centromeric DNA, with docking and confidence scores of −248.4 and 0.87, respectively. Additionally, we observed that the CENPC motif directly interacts with centromeric DNA, while adjacent DNA-binding regions may facilitate the binding of the CENPC motif to DNA (Figure 3C). Subsequently, upon deletion of both the CENPC motif and DNA-binding regions, docking and confidence scores decreased to −171.8 and 0.60, respectively (Figure 3D, Supplementary Figure S1D). Thus, it is likely that the CENPC motif and adjacent DNA-binding regions play a crucial role for the interaction with centromeric DNA.

### CENPC-k and CENPC motifs together with their DNA-binding regions can target centromeres

**Figure 4.**
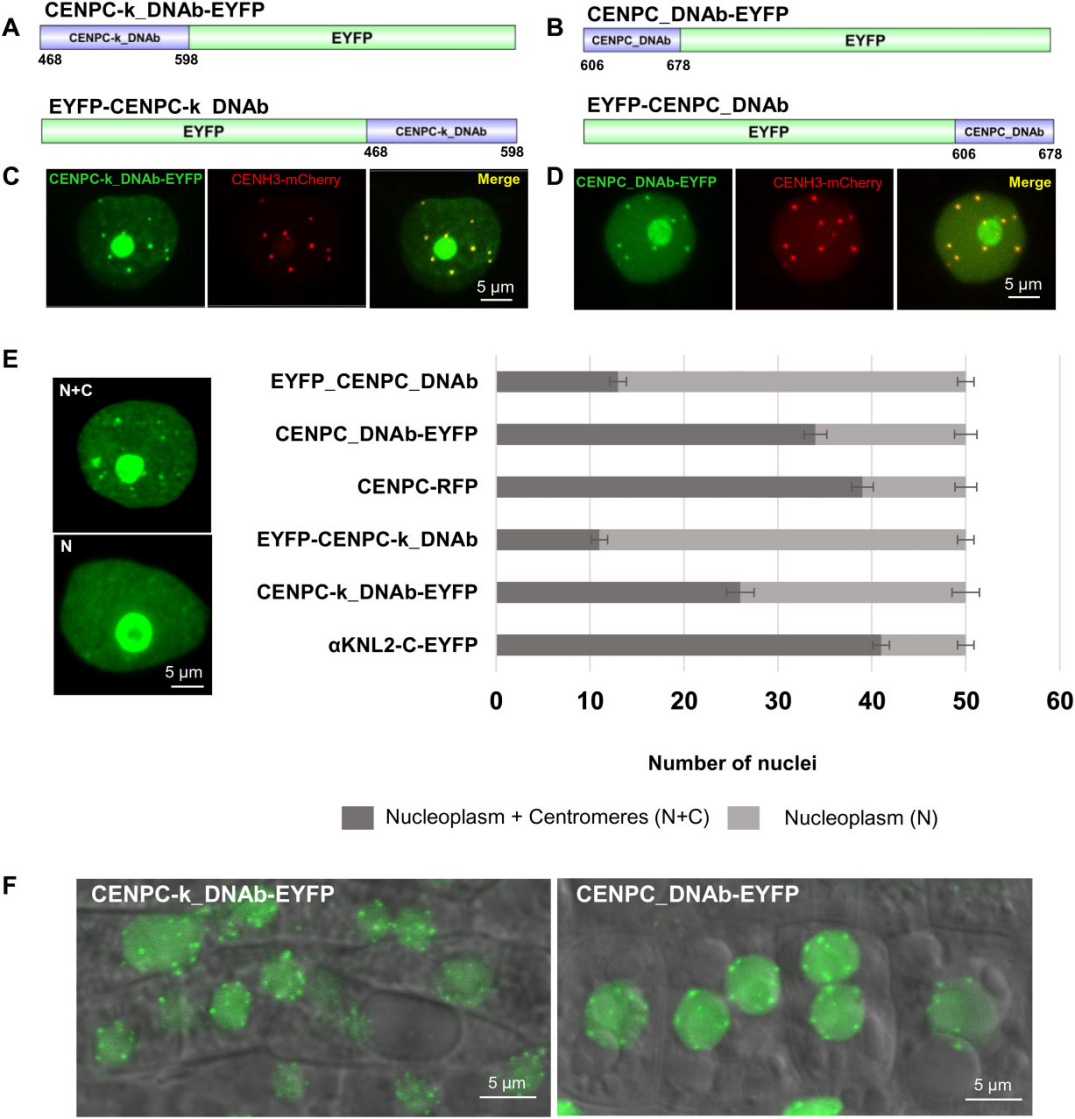
The αKNL2 and CENP-C protein fragments, which include the CENPC-k or CENPC motifs and DNA-binding sites, are capable of targeting centromeres. Fusion constructs of (**A**) CENPC-k_DNAb-EYFP (upper), EYFP-CENPC-k_DNAb (lower) and (**B**) CENPC_DNAb-EYFP (upper), EYFP-CENPC_DNAb (lower). (**C**and**D**) A representative image showing the subcellular localization of the corresponding fusion proteins in the nucleoplasm, at the presumed centromeres, and in the nucleoli of transiently transformed *N. benthamiana*. Co-infiltration with the CENH3-mCherry construct confirmed centromeric localization. (**E**) Distribution of fluorescence patterns in *N. benthamiana* nuclei transformed with constructs containing CENPC-k and CENPC with DNA-binding motifs fused to EYFP. αKNL2-C-EYFP and CENPC-RFP served as positive controls. Two fluorescence patterns were defined: nucleoplasmic and centromeric (N + C), and nucleoplasmic only (N). The frequency of nuclei displaying these patterns was determined for each construct based on 150 nuclei in *N. benthamiana* leaves. Data are presented as mean ± SEM (*n* = 3). (**F**) Representative image showing subcellular localization of CENPC-k_DNAb-EYFP (left) and CENPC_DNAb-EYFP (right) fusion proteins in the nucleoplasm and at presumed centromeres of root tip nuclei of stably transformed *A. thaliana*.

To test whether adding DNA-binding regions to the CENPC-k or CENPC motifs would enhance their centromere-targeting efficiency, constructs containing either the αKNL2 fragment with the CENPC-k motif and its putative DNA-binding regions (CENPC-k_DNAb) or the CENP-C fragment with the CENPC motif and its putative DNA-binding regions (CENPC_DNAb) were generated and fused to EYFP in both orientations (Figure 4A and B).

Initially, all four constructs were transiently expressed in *N. benthamiana*. The *in vivo* fluorescence localization analysis in young *N. benthamiana* leaves showed that the addition of DNA-binding regions to the CENPC-k or CENPC motifs resulted in centromere-specific signals, along with fluorescence distributed in the nucleoplasm (Figure 4C and D). Interestingly, fluorescent signals were consistently detected in the nucleoli of most nuclei for all constructs. In contrast, fluorescence within the nucleolus was rarely observed when expressing the CENPC-k and CENPC EYFP fusion constructs in *N. benthamiana* (Figure 1G and H). This led us to speculate that the inclusion of nucleic acid-binding regions may have facilitated the interaction with RNA. Indeed, both αKNL2 and CENP-C exhibited the capability to bind RNA (12,22). To confirm the centromeric localization of the EYFP-tagged CENPC-k_DNAb and CENPC_DNAb protein fragments, two complementary experiments were conducted. First, *N. benthamiana* leaves were co-infiltrated with constructs expressing either CENPC-k_DNAb or CENPC_DNAb fused to EYFP, along with *Arabidopsis* CENH3 fused to mCherry, which served as a centromere marker. In the second experiment, nuclei from *N. benthamiana* plants infiltrated with the EYFP-tagged CENPC-k_DNAb or CENPC_DNAb constructs were immunostained with antibodies against *N. benthamiana*CENH3. Results from both experiments demonstrated that the EYFP-tagged CENPC-k_DNAb and CENPC_DNAb fragments colocalized with CENH3-mCherry and *N. benthamiana* CENH3 at the centromeres, confirming their centromeric localization in *N. benthamiana* (Figure 4C and D, Supplementary Figure S2A and B). Next, we further analyzed the distribution of fluorescent signals in 150 (3 × 50) nuclei of *N. benthamiana* for each fusion construct. αKNL2-C-EYFP and CENPC-RFP constructs served as positive controls. In all cases, localization patterns in nucleoplasm and at centromeres (N + C) and only in nucleoplasm (N) were observed. Quantitative analyses (Figure 4E) were carried out by evaluating the co-localization of EYFP signals with immunosignals of *N. benthamiana* CENH3 at centromeres. Comparable centromere-targeting efficiency was observed between the αKNL2-C and CENPC-k_DNAb-EYFP fragments, as well as between the CENP-C and CENPC_DNAb-EYFP fragments. However, the EYFP-CENPC-k_DNAb and EYFP-CENPC_DNAb fragments demonstrated reduced efficiency in targeting centromeres (Figure 4E).

To further determine whether sequences between the DNA-binding regions and the CENPC-k motif (amino acids 477–538 and 573–580) in the 468–598 aa fragment of αKNL2 (Figure 1A) affect its centromeric targeting, these regions were deleted using site-directed mutagenesis. The resulting fragment was fused to EYFP and transiently expressed in *N. benthamiana*. Examination of 150 nuclei (3 × 50) revealed that the number of nuclei exhibiting centromeric localization was similar to that observed with the complete 468–598 aa fragment (Supplementary Figure S2C and D). Finally, to analyze the ability of CENPC-k_DNAb/CENPC_DNAb constructs to target *Arabidopsis* centromeres, all four constructs were stably transformed in *Arabidopsis* plants. At least 10 independent transgenic lines were analyzed for each construct. In all cases, fluorescent signals were distributed in the nucleoplasm and at presumed centromeres (Figure 4F). Double immunostaining experiments with anti-GFP and anti-CENH3 antibodies in root tip nuclei of transformants confirmed the centromeric localization of EYFP-tagged fragments (Supplementary Figure S3). Thus, the addition of DNA-binding regions to the CENPС-k and CENPC motifs results in efficient centromere localization.

### Deletion of DNA-binding regions adjacent to the CENPC-k motif reduces centromeric localization of αKNL2-C

**Figure 5.**
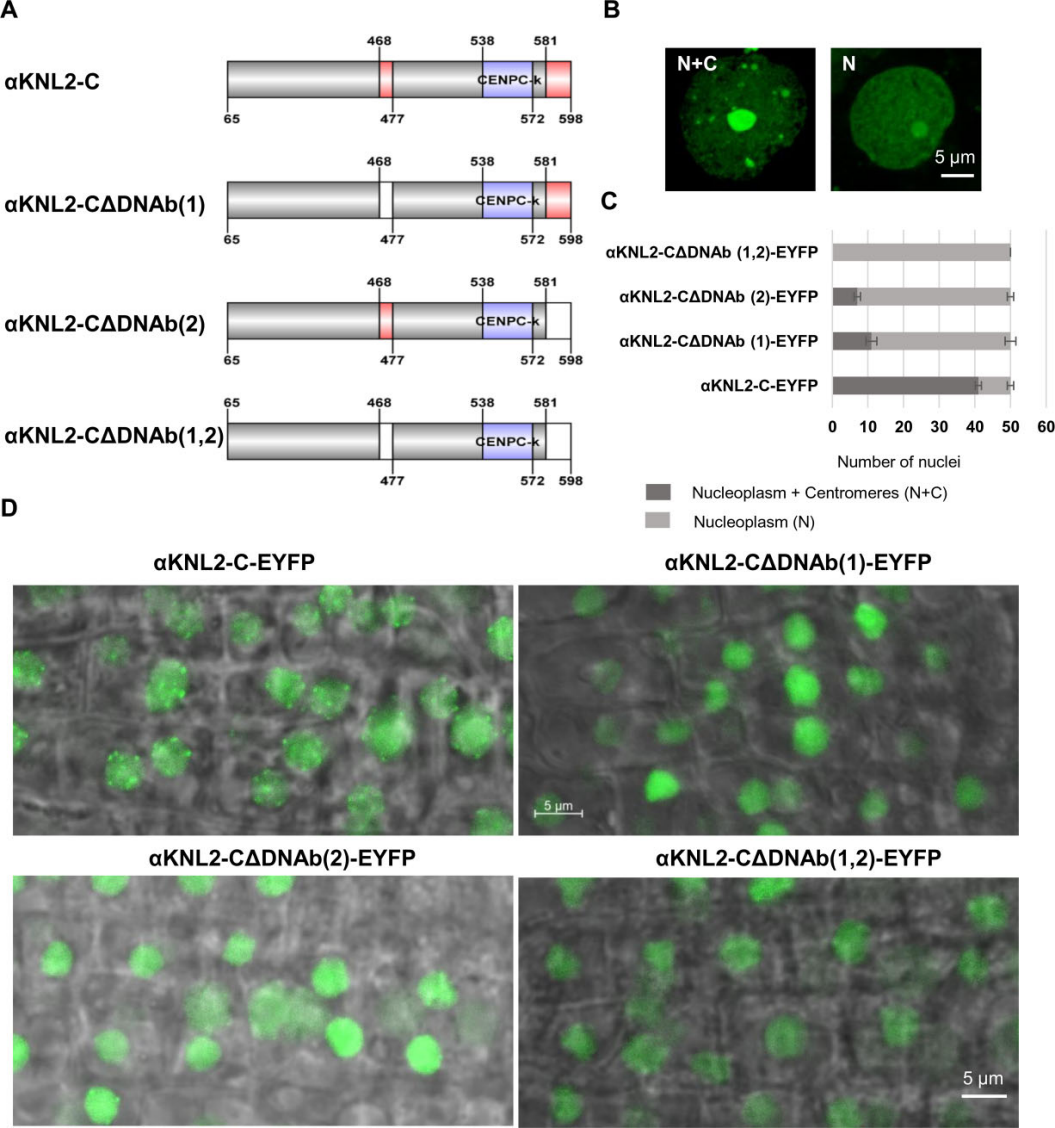
Deletion of DNA-binding regions resulted in reduced centromeric targeting of αKNL2-C in *N. benthamiana* and *A. thaliana*. (**A**) Schematic representation of the C-terminal part of αKNL2 and constructs with deletions of one or two DNA-binding sites. (**B**) *N. benthamiana* nuclei displaying fluorescent signals in the nucleoplasm, at presumed centromeres and in the nucleolus (N + C) or in the nucleoplasm and nucleolus (N). (**C**) Bar chart showing the distribution of nuclei with these localization patterns (N + C and N) in *N. benthamiana* leaves expressing αKNL2-C constructs with deletions of DNA-binding regions, compared to a non-mutagenized control. Data are presented as mean ± SEM (*n* = 3). (**D**) Localization patterns of αKNL2-C protein fragments with deletions of DNA-binding region and a non-mutagenized control in root tips of stably transformed *A. thaliana*.

We demonstrated that the two DNA-binding regions adjacent to the CENPC-k motif can target *N. benthamiana* and *A. thaliana* centromeres. Therefore, to separately investigate the impact of these regions on the centromeric localization of αKNL2, constructs encoding the C-terminal part of αKNL2 lacking one or both DNA-binding regions were generated and designated as αKNL2-CΔDNAb(1)-EYFP, αKNL2-CΔDNAb(2)-EYFP and αKNL2-CΔDNAb(1,2)-EYFP, respectively (Figure 5A). The C-terminus was chosen instead of the full-length αKNL2 as the latter cannot be expressed in plants possibly due to its proteolytic degradation (7). All constructs were transiently expressed in *N. benthamiana*, revealing that when one of the two DNA-binding regions was deleted, localization was observed either in the nucleoplasm and presumed centromeres or solely in the nucleoplasm (Figure 5B). The centromere targeting efficiency of the deletion constructs was compared with that of αKNL2-C. Analysis of 150 (3 × 50) nuclei per construct showed that the number of nuclei with strong centromeric signals was reduced for both deletion constructs compared to the αKNL2-C-EYFP control. It was further observed that the decrease was more pronounced in αKNL2-CΔDNAb(2)-EYFP compared to αKNL2-CΔDNAb(1)-EYFP, which had the largest deletion in the DNA-binding region. In contrast, deletion of both DNA-binding regions almost completely abolished the centromeric localization of the resulting protein (Figure 5C). Stable expression of these three constructs in *A. thaliana* yielded results similar to those obtained for *N. benthamiana* (Figure 5D). At least 10 independent transgenic lines were analyzed for each construct. In the case of the αKNL2-C-EYFP fusion, used as a control, all lines consistently exhibited distinct centromeric signals in the root tip nuclei (Figure 5D, left top panel). In contrast, transgenic lines expressing the mutated constructs showed no clear centromeric signals; instead, strong fluorescence was observed throughout the nucleoplasm, with only occasional centromeric signals visible.

**Figure 6.**
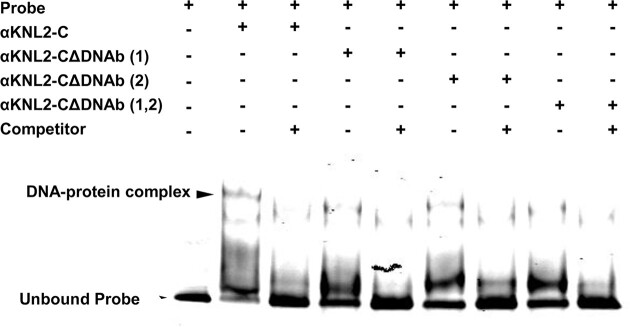
EMSA experiment showing the interaction of αKNL2-C fragments with the centromeric repeat *pAL1*. Labeled *pAL1* repeat probes were incubated with *in vitro* expressed unmodified αKNL2-C protein or protein fragments lacking one of the DNA-binding sites [αKNL2-CΔDNAb(1)], [αKNL2-CΔDNAb(2)] or both sites together [αKNL2-CΔDNAb(1,2)]. Competitor experiments with unlabeled *pAL1* demonstrated specific binding to the *pAL1* DNA probes. The positions of the unbound *pAL1* probe and DNA-protein complexes are indicated by arrows.

In addition, EMSA experiments were conducted with αKNL2-C, αKNL2-CΔDNAb(1), αKNL2-CΔDNAb(2) and αKNL2-CΔDNAb(1,2) proteins expressed with TNT SP6 High Yield Wheat Germ and centromeric DNA probe labeled with IR-dye680 (Supplementary Figure S4A). Previously, we demonstrated that complete deletion of the CENPC-k motif did not influence the ability of αKNL2 to interact with DNA (12). Initially, we confirmed the interaction between non-mutated αKNL2 (αKNL2-C) and the labeled centromeric *pAL1* probe. Indeed, the addition of the unlabeled competitive *pAL1* DNA caused a weaker DNA shift, confirming specific binding. In contrast to the non-mutated αKNL2-C construct, the αKNL2-C protein fragments with deleted DNA-binding regions showed a reduced *pAL1*-binding ability, as indicated by the increased amount of unbound probe when one or both DNA-binding motifs were deleted (Figure 6, Supplementary Figure S4B).

### The CENPC-k motif combined with a non-plant DNA-binding region can target centromeres

**Figure 7.**
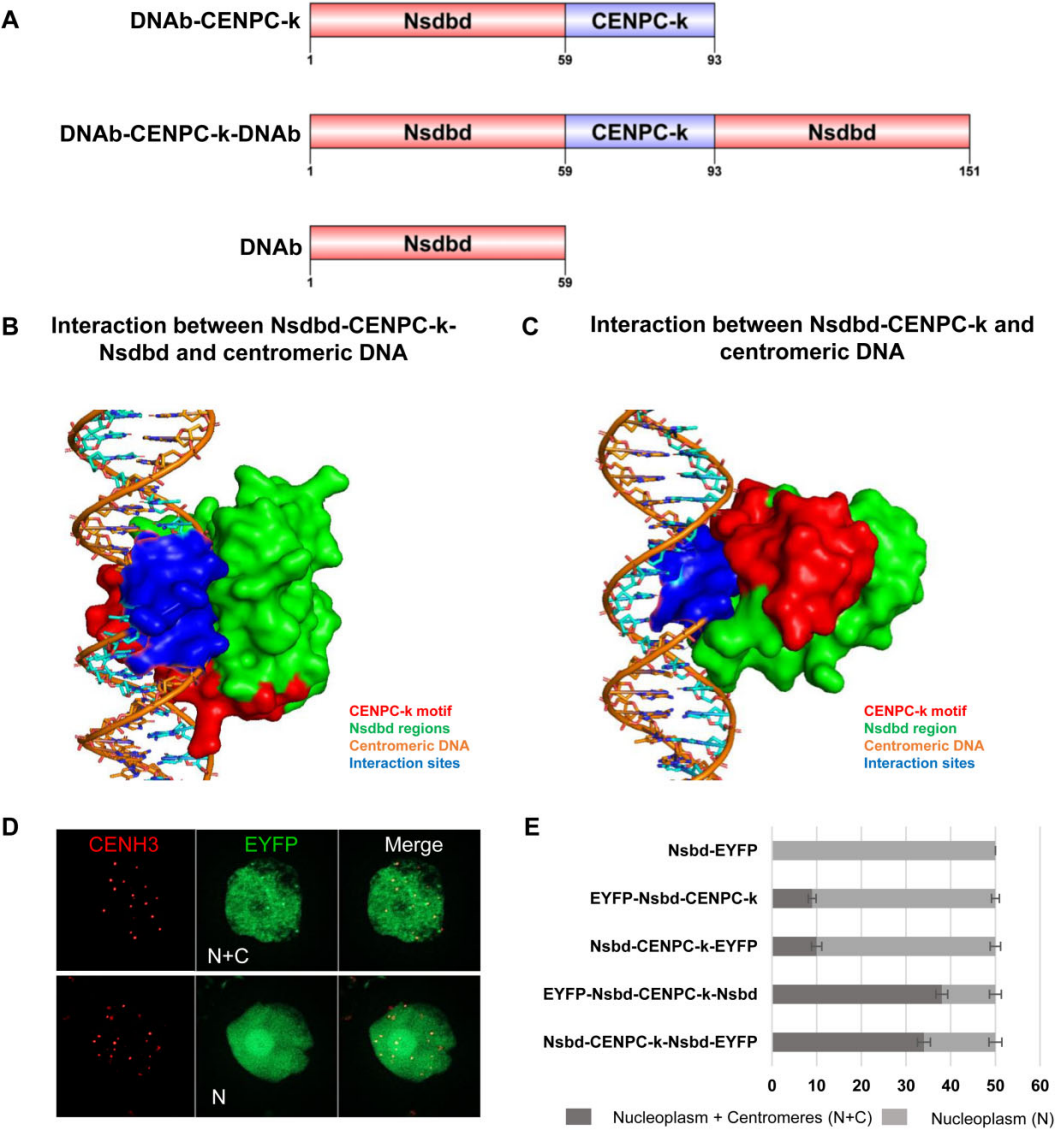
The CENPC-k motif, combined with bacterial DNA-binding regions, can target centromeres in *N. benthamiana*. (**A**) Schematic representation of fragments containing the CENPC-k motif and bacterial DNA-binding regions (Nsdbd), as well as the DNA-binding motif alone, used as a negative control. (**B**) Docking simulations reveal that the Nsdbd-CENPC-k-Nsdbd protein fragment interacts with centromeric DNA (*pAL1*) through the Nsdbd regions. (**C**) A reduced interaction with *pAL1* was observed when the Nsdbd DNA-binding region was fused only to the N-terminal part of CENPC-k. The protein is shown in surface and the DNA in sticks models. (**D**) Distribution of fluorescence patterns in *N. benthamiana* nuclei infiltrated with constructs containing CENPC-k in combination with DNA-binding region of bacteria or DNA binding region alone in fusion with EYFP. Two fluorescence patterns were defined: nucleoplasmic and centromeric (N + C), and nucleoplasmic only (N). Immunostaining with *N. benthamiana* CENH3 antibodies confirmed centromeric localization. (**E**) Frequency of nuclei displaying these fluorescence patterns was determined for each construct based on 150 nuclei in *N. benthamiana* leaves. Data are presented as mean ± SEM (*n* = 3).

So far, our findings indicate that the DNA-binding regions of αKNL2 confer DNA-binding, while the CENPC-k motif ensures the centromere-specificity of this DNA affinity. Given that DNA-binding regions exhibit lower conservation compared to the CENPC-k motif but share a positive charge characteristic, we hypothesized that, regardless of its sequence, any DNA-binding motif could promote centromere localization when combined with CENPC-k. In *E. coli*, the abundant H-NS is known to bind chromosomal DNA at numerous sites, playing a crucial role in bacterial nucleoid organization (40). The H-NS protein comprises an N-terminal dimerization domain and a C-terminal Nsdbd separated by a linker region (41). We decided to test whether the Nsdbd domain would lead to the anchoring of the CENPC-k motif on centromeres, similar to the endogenous DNA-binding motifs of αKNL2. Therefore, the Nsdbd was fused to the N-terminus of CENPC-k (Nsdbd-CENPC-k) or flanked on both sides simultaneously (Nsdbd-CENPC-k-Nsdbd) (Figure 7A). The resulting 3D structures of these fragments were modeled (Supplementary Figure S5A and B), and a molecular docking analysis of Nsdbd-CENPC-k and Nsdbd-CENPC-k-Nsdbd with centromeric DNA was conducted. Interestingly, the construct with CENPC-k flanked by two Nsdbd exhibited a stronger affinity for centromeric DNA compared to the single Nsdbd-CENPC-k fusion, with docking and confidence scores of −210.31 and 0.76 and −178.53 and 0.63, respectively (Figure 7B, Supplementary Figure S5C).

To validate our molecular docking results, Nsdbd-CENPC-k and Nsdbd-CENPC-k-Nsdbd fragments were fused to EYFP at either the N- or C-termini, and *Agrobacteria* suspensions expressing these constructs were infiltrated into *N. benthamiana* leaves. The control construct, consisting of only the Nsdbd fused to EYFP, displayed homogeneous fluorescent signals throughout the nucleoplasm. In contrast, distinct fluorescent spots, presumably corresponding to centromeres, were observed in many nuclei when two Nsdbds were fused to the CENPC-k motif (Figure 7D and E). Immunostaining with *N. benthamiana* CENH3 antibodies confirmed the centromeric nature of distinct EYFP signals (Figure 7D).

## Discussion

The interaction between inner kinetochore proteins and DNA is crucial for proper chromosome segregation during cell division (42,43). Besides CENH3, CENP-C, CENP-T, CENP-N and αKNL2 are known to interact with DNA (12,18). However, the mechanism of this interaction is not well understood. Here we identify putative DNA-binding regions of αKNL2 and CENP-C proteins from *A. thaliana* and focus on their role and mechanism of centromere targeting in living plant cells.

KNL2 recruitment to centromeres is a critical step for the kinetochore assembly and in the loading pathway of CENH3. The plant αKNL2 protein contains a conserved SANTA domain at the N-terminus and a centromere-targeting CENPC-k motif at the C-terminus (7,12). Fusion of the C-terminal part of αKNL2 (containing the CENPC-k motif but lacking the SANTA domain) with EYFP showed centromeric localization in transiently transformed *N. benthamiana* and stably transformed *A. thaliana* (7). Since deletion of the CENPC-k motif abolishes centromeric localization of αKNL2-C, it was assumed that this motif plays an important role in targeting αKNL2 to centromeres. The importance of the CENPC-k motif for centromere targeting has been demonstrated for the KNL2 homologues from chicken (15) and frog (16). Originally, the CENPC motif has been identified and described as a typical feature of the CENP-C protein (44,45). It is required to direct CENP-C to centromeric nucleosomes in human cells (46–48). A mutation of the conserved amino acids in the rat CENPC motifs disrupted its ability to bind to centromeric nucleosomes (Kato *et al.* 2013). Interestingly, the abolished centromeric localization of αKNL2-C, caused by deletion of the CENPC-k motif, can be restored by inserting the corresponding motif from *Arabidopsis* CENP-C (12).

Here, we showed that the CENPC motifs of αKNL2 and CENP-C alone are insufficient to target centromeres in *N. benthamiana* and *A. thaliana*. Fusion of either CENPC-k or CENPC with EYFP resulted in fluorescent signals in nucleoplasm and cytoplasm but no centromeric localization, neither in transiently transformed *N. benthamiana* nor in stably transformed *A. thaliana*. Previous studies have shown that αKNL2 of *A. thaliana* (12), CENP-C of human (23) and maize (22) can interact with DNA in a sequence-independent manner. Hence, we hypothesized that the DNA-binding capability might contribute to stabilizing KNL2 and CENP-C at centromeres. Our investigation identified positively charged regions flanking both sides of the CENPC-k and CENPC motifs of the αKNL2 and CENP-C proteins, respectively, in all selected for analysis plant species (Figure 2), although no well-defined DNA-binding domains were found. Interestingly, these regions showed lower sequence conservation across plant species compared to the CENPC-like motifs. Supporting our hypothesis, fragments of αKNL2 or CENP-C protein containing the CENPC-like motif and putative DNA-binding regions in fusion with EYFP exhibited centromeric localization in both transiently transformed *N. benthamiana* and stably transformed *Arabidopsis*.

**Figure 8.**
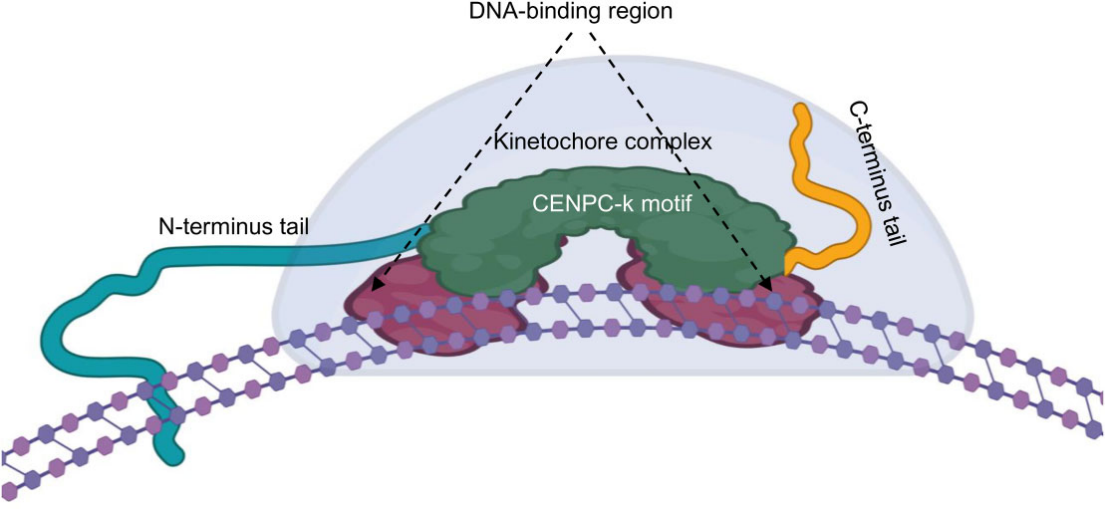
Mechanism of anchoring of kinetochore complex proteins to the centromere through DNA-binding regions. The centromeric anchoring of kinetochores relies on the CENPC-k motif and is reinforced by adjacent, sequence-independent DNA-binding regions, ensuring stable interaction with centromeric DNA. Created using BioRender.com.

Deletion of one of the DNA-binding regions from the C-terminal part of αKNL2 reduced its centromeric localization, while deletion of both DNA-binding regions abolished it completely despite the presence of the centromere targeting CENPC-k motif. Our experimental data were also confirmed by the bioinformatic prediction of the αKNL2-DNA interaction model, which is consistent with the model presented in our complementary study predicting the interaction of KNL2 variants with the centromeric nucleosome (49). We conclude that targeting of αKNL2 to centromeres requires the conserved CENPC-k motif, but its anchoring at centromeres depends on the less conserved DNA-binding regions (Figure 8). Therefore, we postulate that the fusion of the CENPC-k motif with any DNA-binding sites capable of binding DNA in a sequence-independent manner would result in targeting the centromere. Indeed, fusion of CENPC-k to the Nsdbd of a bacterial H-NS protein (40) promoted its centromeric localization in *N. benthamiana*.

Furthermore, using online tools, M18BP1/KNL2 sequences from various vertebrates and *C. elegans*, as well as α-, β-, γ- and δKNL2 variants from plants were analyzed. All proteins tested demonstrated the ability to bind to DNA/RNA nucleic acids as shown in Table 1. Notably, DNA-binding regions were identified in KNL2 variants both with (α- and γ-KNL2 of plants, M18BP1 of non-mammalian vertebrates) and without (β- and δ-KNL2 of plants, M18BP1 of mammals) the CENPC motif. Based on this analysis, we suggest that M18BP1/KNL2 proteins across different organisms may employ a similar centromere-targeting mechanism, with DNA-binding sites playing a crucial role in their anchoring to centromeres. This highlights the need for further investigation into KNL2’s centromere-targeting function across diverse taxa.

In our recent study (49), we provided experimental evidence confirming the direct interaction between βKNL2 and centromeric DNA, thereby supporting the accuracy of prior predictions. Although βKNL2 targeting is dependent on αKNL2 in certain tissues, our AlphaFold2 based model predicts that both KNL2 variants can bind to centromeric nucleosome DNA independently of each other (49).

Recently Ariyoshi *et al.* (50) demonstrated that the CM peptide (643–683 aa) of chicken CENP-C, including the CENPC motif (655–675 aa), binds the CENP-A nucleosome *in vitro* but lacks the stability and specificity of the full C-terminal region. Similarly, the KNL2 fragment (518–560 aa) of chicken KNL2, containing the CENPC-like motif, is essential for recognizing centromeric CENP-A nucleosomes (17) but requires upstream regions for efficient and stable binding. These findings align with our results, indicating that the CENPC-like motifs require adjacent regions for effective binding to centromeric nucleosomes. However, whether these regions in chicken KNL2 and CENP-C possess DNA-binding properties remains to be clarified.

The ability of kinetochore assembly proteins (e.g., KNL2/M18BP1, CENP-C) to interact with DNA and RNA in a sequence-independent manner, as summarized in the review by Ramakrishnan Chandra *et al.* (51), likely enables these proteins to adapt to the rapid evolution of centromeric sequences. This adaptability might facilitate the formation of functional kinetochores in newly established centromeric regions (neocentromeres) when the original centromere becomes inactive. Moreover, it may allow these proteins to recognize centromeres across species, a feature particularly relevant in the context of intra- and interspecific hybridization. The successful localization of the αKNL2-C and CENP-C proteins from *Arabidopsis*, along with their fragments containing the CENPC-k or CENPC motif and DNA-binding regions, to the centromeres of *N. benthamiana*- despite substantial differences in centromeric DNA sequences between these species - supports this hypothesis.

In summary, our study reveals that the centromere-targeting motifs CENPC-k and CENPC require adjacent sequence-independent DNA-binding regions to achieve effective centromeric localization. This mechanism likely represents an evolutionary adaptation that confers flexibility and robustness to kinetochore assembly, allowing it to operate effectively across diverse and rapidly evolving centromeric DNA landscapes. These findings deepen our understanding of centromere architecture and open new opportunities for synthetic biology and chromosomal engineering, with potential for manipulating centromere identity and kinetochore function in plant and animal genomes.

Future research could utilize the identified centromere-targeting unit to guide chromatin-modifying proteins to the centromere, allowing for the assessment of how chromatin modifications influence kinetochore assembly and function. To gain structural insights, techniques, such as cryo-EM and molecular dynamics simulations could visualize plant-specific interactions between KNL2 variants (α- and βKNL2), CENP-C and CENP-A nucleosomes, supporting the hypothesis of conserved but plant-adapted centromere-recognition mechanisms. Finally, the application of functional genomics, such as CRISPR-mediated mutagenesis of DNA-binding regions in plants and other model systems, might provide critical insights into the precise mechanisms by which αKNL2 and CENP-C localize to the centromere and facilitate kinetochore assembly and function.

## Supplementary Material

gkae1242_Supplemental_Files

## Data Availability

All relevant data are within the manuscript and its supporting information files.
