# Supplementary material for: Centromeric localization of αKNL2 and CENP-C proteins in plants depends on their centromere-targeting domain and DNA-binding regions
Source: Nucleic Acids Res. 2024 Dec 24;53(4):gkae1242. doi: 10.1093/nar/gkae1242 (PMC11879092; doi:10.1093/nar/gkae1242)
Supplement: gkae1242_Supplemental_Files [file gkae1242_supplemental_files.zip › Supplementary Figures_1-5.pdf]

A B

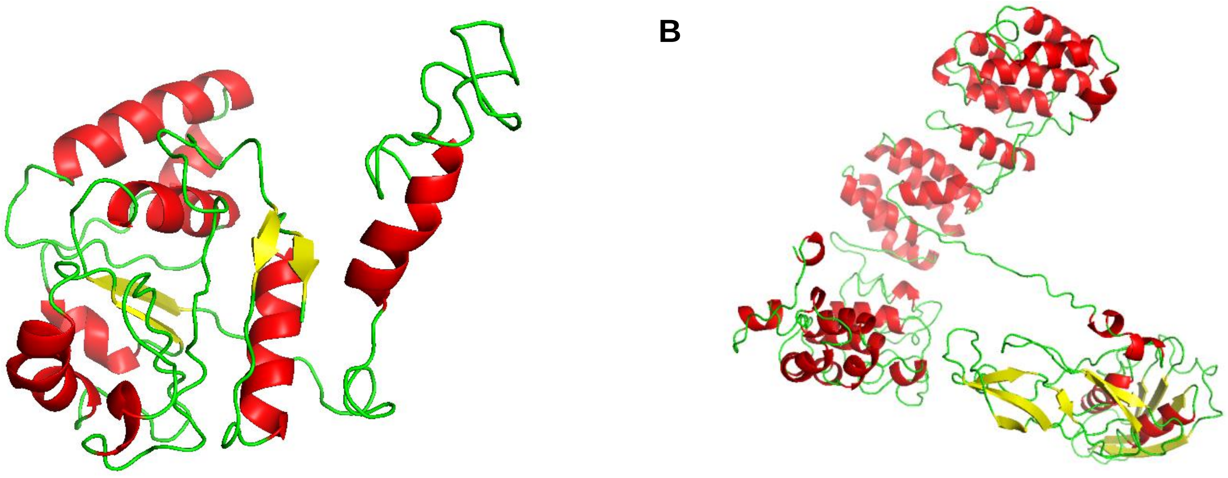

C

Rank of the predicted model	1	2	3
**αKNL2-C-*pAL1***			
Docking Score	-238.2	-231.9	-225.9
Confidence Score	0.85	0.83	0.82
**αKNL2-CΔDNAb(1,2)-*pAL1***			
Docking Score	-179.1	-165.7	-163.0
Confidence Score	0.53	0.52	0.51

D

Rank of the predicted model	1	2	3
**CENP-C-*pAL1***			
Docking Score	-248.4	-225.2	-224.4
Confidence Score	0.87	0.81	0.81
**CENP-C-ΔCENPC-DNAb(1,2)-*pAL1***			
Docking Score	-171.8	-170.1	-169.6
Confidence Score	0.60	0.59	0.59

Supplementary Figure 1. The three-dimensional models of αKNL2-C and CENP-C proteins and their interaction scores with *pAL1*. **(A, B)** The three-dimensional structure of αKNL2-C **(A)** and CENP-C **(B)** proteins predicted by I-TASSER. **(C, D)** Tables showing the docking and confidence scores for the top 3 prediction models of αKNL2-C and its variant αKNL2-CΔDNAb(1,2) **(C)** as well as CENP-C and its truncated form CENP-C-ΔCENPC-DNAb(1,2) **(D)** interaction with *pAL1.*

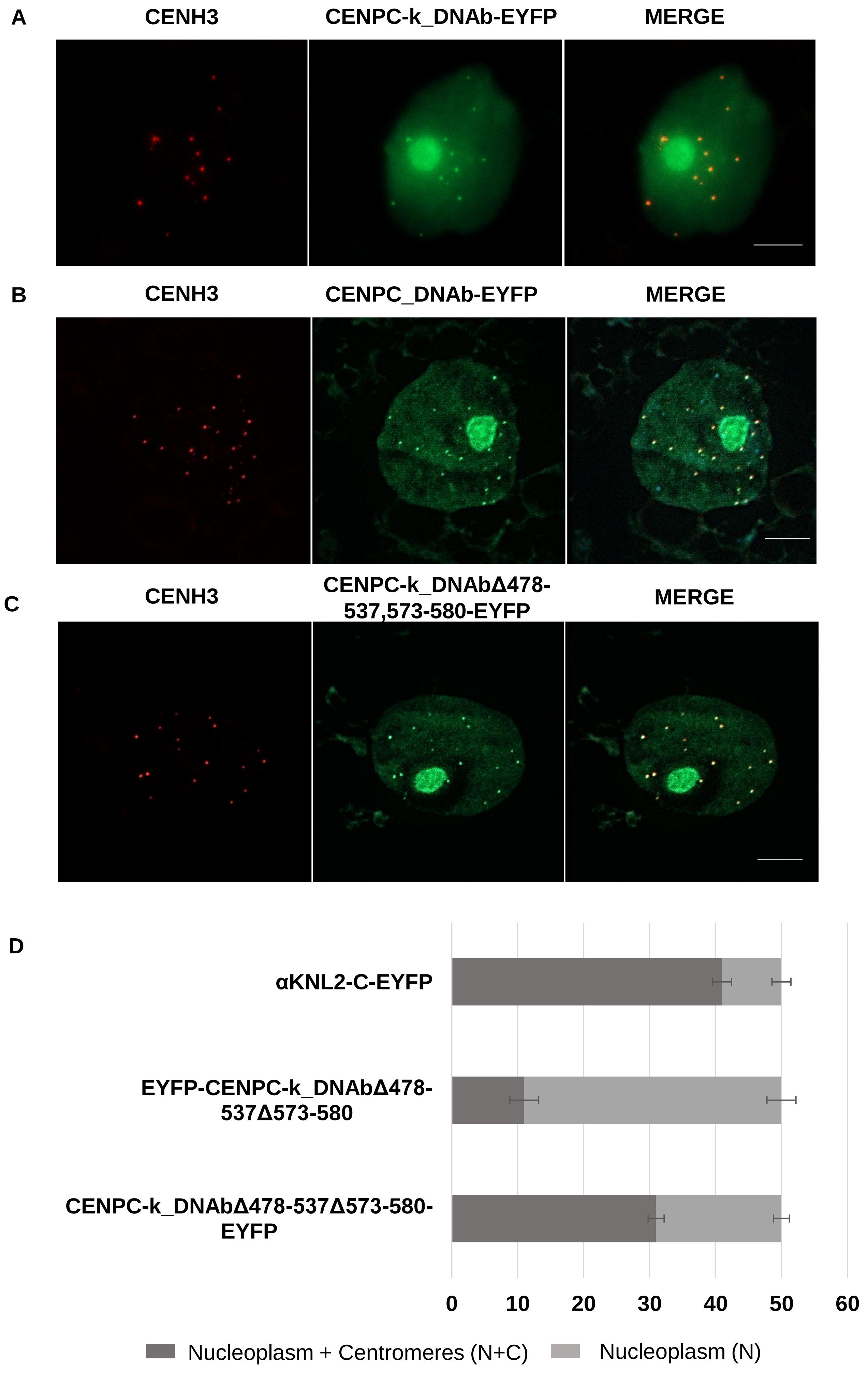
A
CENH3
CENPC-k_DNAb-EYFP
MERGE
B
CENH3
CENPC_DNAb-EYFP
MERGE
C
CENH3
CENPC-k_DNAbΔ478-537,573-580-EYFP
MERGE
D
αKNL2-C-EYFP
EYFP-CENPC-k_DNAbΔ478-537Δ573-580
CENPC-k_DNAbΔ478-537Δ573-580-EYFP
0
10
20
30
40
50
60
Nucleoplasm + Centromeres (N+C)
Nucleoplasm (N)

Supplementary Figure 2. Colocalization of EYFP signals with *N. benthamiana* CENH3 in nuclei of transiently transformed *N. benthamiana* leaves. **(A-C)** Representative images illustrating the subcellular localization of the fusion proteins: **(A)** CENPC-k_DNAb-EYFP, **(B)** CENPC_DNAb-EYFP, and **(C)** CENPC-k_DNAbΔ478-537,Δ573-580-EYFP. Fluorescence signals were observed in the nucleoplasm, at the presumed centromeres, and within the nucleoli of transiently transformed *N. benthamiana* nuclei. Centromeric localization was confirmed via immunostaining using an *N. benthamiana* CENH3-specific antibody. **(D)** Quantitative analysis of fluorescence patterns in nuclei expressing the fusion proteins CENPC-k_DNAbΔ478-537,Δ573-580-EYFP and EYFP-CENPC-k_DNAbΔ478-537,Δ573-580 compared to the control construct αKNL2-C-EYFP. Two distinct fluorescence patterns were classified: N+C: Nucleoplasmic and centromeric localization, N: Nucleoplasmic localization only. The frequency of nuclei exhibiting these two patterns was determined for each construct, based on the analysis of 150 nuclei in transiently transformed *N. benthamiana* leaves.

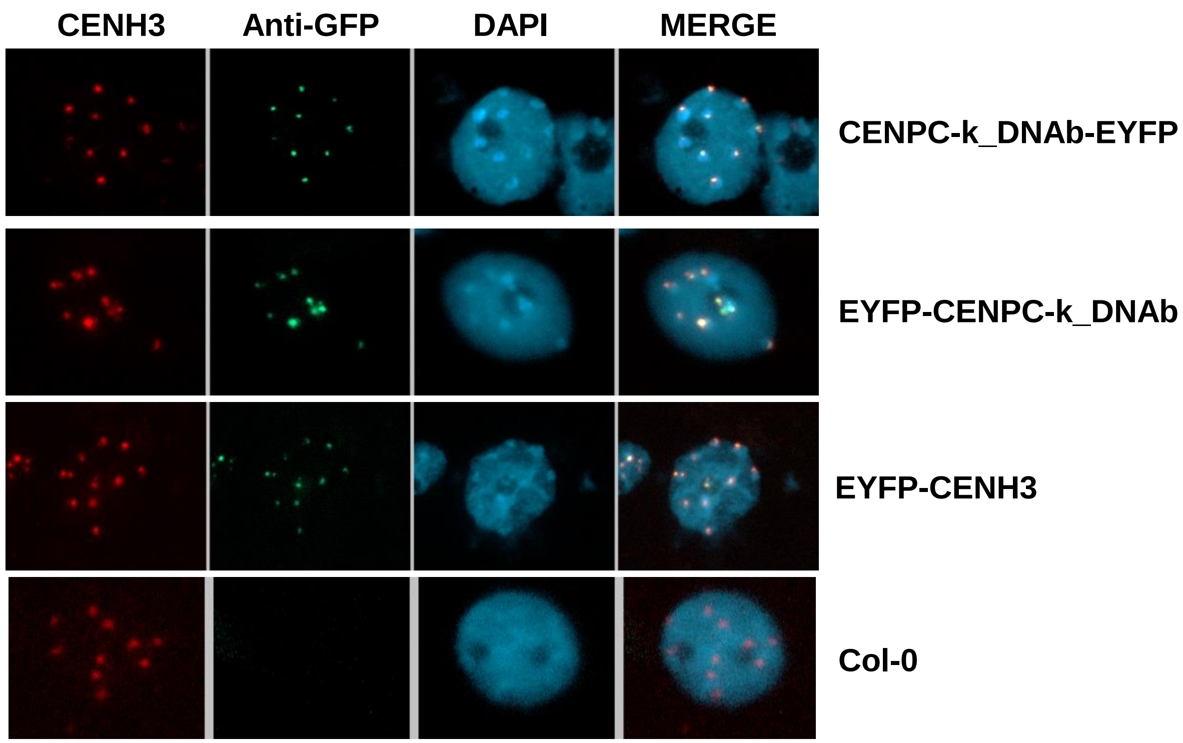

Supplementary Figure 3. Double immunolabeling of *A. thaliana* root tip meristem nuclei using anti-GFP and CENH3 antibodies. To confirm the centromeric localization of CENPC-k_DNAb fragments fused to EYFP in transgenic *A. thaliana* plants, double immunostaining on root tip nuclei of both CENPC-k_DNAb-EYFP and EYFP-CENPC-k_DNAb transgenic plants was performed using anti-CENH3 and anti-GFP antibodies, which also recognize EYFP protein (Chromotek). EYFP-CENH3 transgenic plants (Lermontova et al., 2006) were used as a positive control for the anti-GFP antibodies, while Col-0 plants served as the negative control.

A

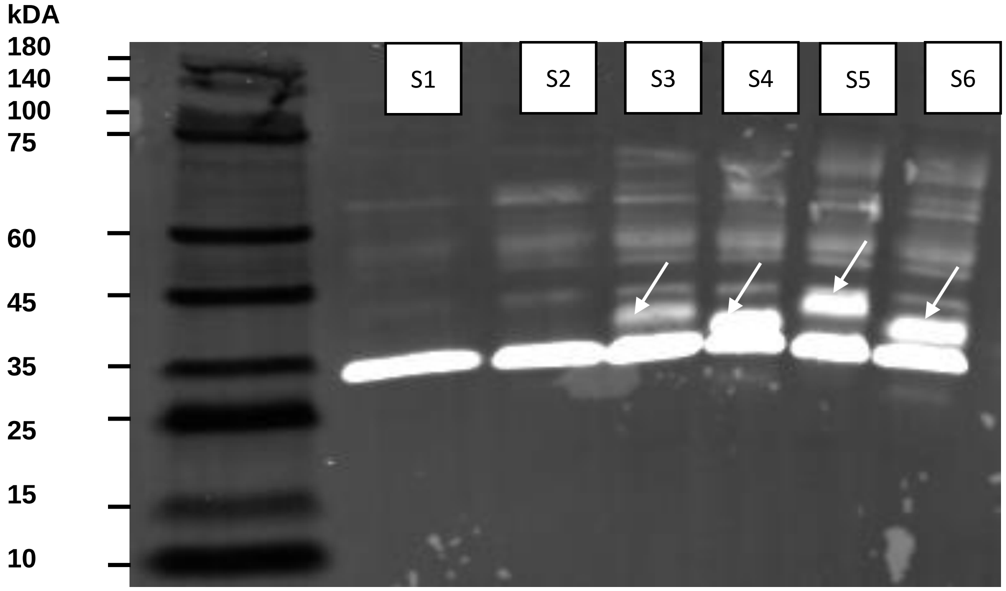

Protein ladder : 180kDa
S1 and S2 - (-ve control, wheat germ reaction without protein expressing plasmid)
S3 - αKNL2-C
S4 - αKNL2-CΔDNAb(1)
S5 - αKNL2-CΔDNAb(2)
S6 - αKNL2-CΔDNAb(1,2)

B

**Probe**	+	+	+	+	+	+	+	+	+
**αKNL2-C**	-	+	+	-	-	-	-	-	-
**αKNL2-CΔDNAb(1)**	-	-	-	+	+	-	-	-	-
**αKNL2-CΔDNAb(2)**	-	-	-	-	-	+	+	-	-
**αKNL2-CΔDNAb(1,2)**	-	-	-	-	-	-	-	+	+
**Competitor**	-	-	+	-	+	-	+	-	+

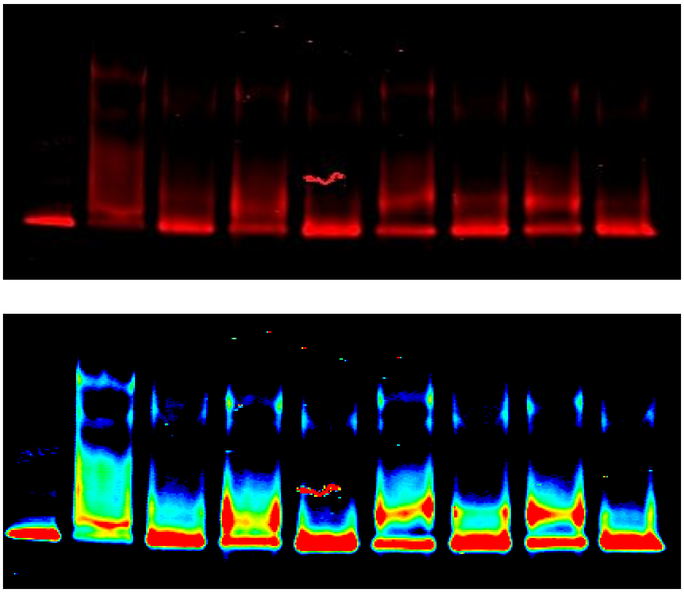

Supplementary Figure 4. Western blot analysis to validate the expression of αKNL2-C protein and its mutated variants with deletions of DNA-binding sites in TNT SP6 High Yield Wheat Germ and EMSA experiment (additional to Figure 6). **(A)** The pF3A WG (Promega) vectors containing the cDNAs of αKNL2-C, αKNL2-CΔDNAb(1), αKNL2-CΔDNAb(2) and αKNL2-CΔDNAb(1,2) fused with a FLAG tag were used for protein expression with TNT SP6 High Yield Wheat Germ system (Promega). Expression of proteins was validated by performing a Western blot analysis against the FLAG-epitope tag using an anti-FLAG antibody. Arrows show αKNL2-C protein variants expressed in TNT SP6 High Yield Wheat Germ system. Estimated weight of protein fragments: αKNL2-C - 26,3 kDa; αKNL2-CΔDNAb(1) – 24,8 kDa, αKNL2-CΔDNAb(2) – 27,6 kDa, αKNL2-CΔDNAb(1,2) – 23,4 kDa. Generation of an αKNL2-CΔDNAb(2) expressing clone by mutagenesis resulted in deletion of the stop codon and inclusion of additional amino acids encoded by the vector sequence, leading to an increase in the weight of the corresponding protein fragment from 24.4 to 27.6 kDa. **(B)** The EMSA images from Figure 6 are presented here with increased contrast for improved visualization. In the upper panel, the images are artificially colored red to enhance contrast. The bottom panel shows the corresponding saturation images. Together, these modifications accentuate the differences in the amount of unbound probe between samples, allowing for clearer comparisons.

A **B**

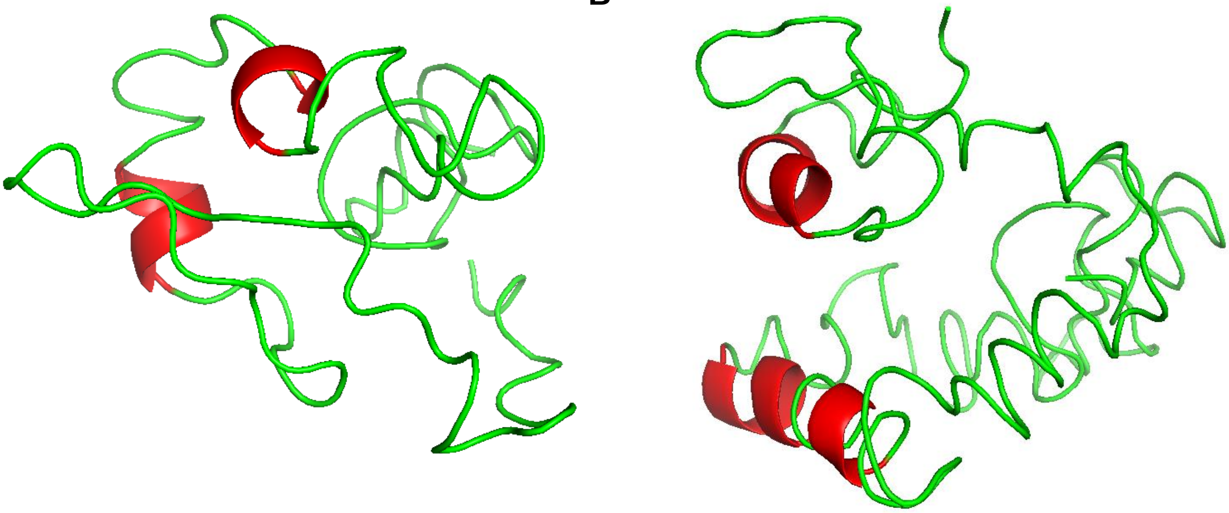

C

**Rank of the predicted model**	**1**	**2**	**3**
**Nsdbd-CENPC-k-Nsdbd-*pAL1***			
Docking Score	-210.31	-197.15	-193.08
Confidence Score	0.76	0.71	0.70
**Nsdbd-CENPC-k-*pAL1***			
Docking Score	-178.53	-176.49	-171.89
Confidence Score	0.63	0.62	0.60

Supplementary Figure 5. The three-dimensional models of non-specific DNA binding domain (Nsdbd) with CENPC-k and its interaction score with *pAL1.* **(A, B)** The three-dimensional structure of Nsdbd-CENPC-k **(A)** and Nsdbd-CENPC-k-Nsdbd **(B)** protein predicted by I-TASSER. **(C)** The table showing the docking and confidence scores of top 3 prediction models for Nsdbd-CENPC-k-Nsdbd and Nsdbd-CENPC-k interaction with *pAL1.*
