# Supplementary material for: Centromeric localization of αKNL2 and CENP-C proteins in plants depends on their centromere-targeting domain and DNA-binding regions
Source: Nucleic Acids Res. 2024 Dec 24;53(4):gkae1242. doi: 10.1093/nar/gkae1242 (PMC11879092; doi:10.1093/nar/gkae1242)
Supplement: gkae1242_Supplemental_Files [file gkae1242_supplemental_files.zip › Supplementary Tables 1-4.docx]

**Supplementary Table 1: Nucleotide sequences of CENPC-k and CENPC motifs**

| CENPC-k | **(ATG)**CAGAAACGGTCAAGATCAGGAAGGGTGCTTGTGTCATCACTAGAGTTTTGGCGTAACCAAATTCCTGTTTATGATATGGATCGGAACCTTATCCAAGTAAAG |
| --- | --- |
| CENPC | **(ATG)**GGTGGTGTCAGACGAAGTACAAGGATTAAGTCGAGACCGCTCGAATACTGGAGAGGTGAAAGATTCTTGTACGGACGCATCCATGAGAGTTTGACTACTGTT |

An ATG start codon was introduced in cases of C-terminal fusion with EYFP.

**Supplementary Table 2: Nucleotide sequences of CENPC-k_DNA-binding motif fragments**

**of αKNL2**

| CENPC-k_DNAb | **(ATG)**AAAGGAAAATCAAAGAAAAGTGAGAAGACCCTTCAAAGTAACAGCAATGTTGTAGAGCCTATGAATCATTTCAGGTCTGAAGCTGAAGAAGCTGAAGAAAACTTGTCATGGGAAAAAATAAAGAGGAAAATCGACTTTGATGTGGAGGTAACACCGGAAAAAAAAGTGAAGCAGCAGAAGACCAATGCGGCGTCTACTGATTCATTGGGACAGAAACGGTCAAGATCAGGAAGGGTGCTTGTGTCATCACTAGAGTTTTGGCGTAACCAAATTCCTGTTTATGATATGGATCGGAACCTTATCCAAGTAAAGGATGGTAGTGAGACTAACTCCGCTCCATCTAAAGGAAAAGGATCGGATTCTCGAAAGCGAAGAAACTTGAAAATCAAA |
| --- | --- |
| CENPC-k_DNAbΔ478-537,Δ573-580 | **(ATG)**AAAGGAAAATCAAAGAAAAGTGAGAAGGGACAGAAACGGTCAAGATCAGGAAGGGTGCTTGTGTCATCACTAGAGTTTTGGCGTAACCAAATTCCTGTTTATGATATGGATCGGAACCTTATCCAAGTAAAGCCATCTAAAGGAAAAGGATCGGATTCTCGAAAGCGAAGAAACTTGAAAATCAAA |
| CENPC_DNAb | **(ATG)**AAGAAGCCGAAGAAAACTCTTACGCATGAGGGGAAACTATTCTCTTGCAGGAAAAGCCTTGCTGCTGCTGGTACTAAAATTGAAGGTGGTGTCAGACGAAGTACAAGGATTAAGTCGAGACCGCTCGAATACTGGAGAGGTGAAAGATTCTTGTACGGACGCATCCATGAGAGTTTGACTACTGTTATCGGCATAAAGTATGCATCCCCTGGAGAAGGT |

An ATG start codon was introduced in cases of C-terminal fusion with EYFP.

**Supplementary Table 3: Nucleotide sequences of αKNL2-C deletion constructs**

| **αKNL2-C deletion constructs** | **Sequences of αKNL2-C fragments with deletions of DNA-binding sites** |
| --- | --- |
| αKNL2-CΔDNA(1) | ATGAATTACTCTGGGACGAAAGTCAAAAGTGCGGAAAACAAAAGGAAAATCGATGCGAGTAAACTCCAGAGTCCGACTAGTAATGTTGCAGAACATAGTAAGGAAGGTTTAAATAATGCTAAGAGCAATGACGTAGAAAAAGATGTATGTGTGGCTATCAATAATGAAGTGATATCACCAGTGAAGGGATTTGGTAAAAGGCTTTCTGGTACAGATGTTGAAAGATTAACAAGTAAGAATGCTACTAAAGAATCACTGACGTCAGTACAGCGAAAAGGTAGAGTGAAGGTATCGAAGGCATTTCAAGATCCCCTGTCGACCCTTCAAAGTAACAGCAATGTTGTAGAGCCTATGAATCATTTCAGGTCTGAAGCTGAAGAAGCTGAAGAAAACTTGTCATGGGAAAAAATAAAGAGGAAAATCGACTTTGATGTGGAGGTAACACCGGAAAAAAAAGTGAAGCAGCAGAAGACCAATGCGGCGTCTACTGATTCATTGGGACAGAAACGGTCAAGATCAGGAAGGGTGCTTGTGTCATCACTAGAGTTTTGGCGTAACCAAATTCCTGTTTATGATATGGATCGGAACCTTATCCAAGTAAAGGATGGTAGTGAGACTAACTCCGCTCCATCTAAAGGAAAAGGATCGGATTCTCGAAAGCGAAGAAACTTGAAAATCAAA |
| αKNL2-CΔDNAb(2) | ATGAATTACTCTGGGACGAAAGTCAAAAGTGCGGAAAACAAAAGGAAAATCGATGCGAGTAAACTCCAGAGTCCGACTAGTAATGTTGCAGAACATAGTAAGGAAGGTTTAAATAATGCTAAGAGCAATGACGTAGAAAAAGATGTATGTGTGGCTATCAATAATGAAGTGATATCACCAGTGAAGGGATTTGGTAAAAGGCTTTCTGGTACAGATGTTGAAAGATTAACAAGTAAGAATGCTACTAAAGAATCACTGACGTCAGTACAGCGAAAAGGTAGAGTGAAGGTATCGAAGGCATTTCAAGATCCCCTGTCGAAAGGAAAATCAAAGAAAAGTGAGAAGACCCTTCAAAGTAACAGCAATGTTGTAGAGCCTATGAATCATTTCAGGTCTGAAGCTGAAGAAGCTGAAGAAAACTTGTCATGGGAAAAAATAAAGAGGAAAATCGACTTTGATGTGGAGGTAACACCGGAAAAAAAAGTGAAGCAGCAGAAGACCAATGCGGCGTCTACTGATTCATTGGGACAGAAACGGTCAAGATCAGGAAGGGTGCTTGTGTCATCACTAGAGTTTTGGCGTAACCAAATTCCTGTTTATGATATGGATCGGAACCTTATCCAAGTAAAGGATGGTAGTGAGACTAACTCCGCTCCA |
| αKNL2-CΔDNAb(1,2) | ATGAATTACTCTGGGACGAAAGTCAAAAGTGCGGAAAACAAAAGGAAAATCGATGCGAGTAAACTCCAGAGTCCGACTAGTAATGTTGCAGAACATAGTAAGGAAGGTTTAAATAATGCTAAGAGCAATGACGTAGAAAAAGATGTATGTGTGGCTATCAATAATGAAGTGATATCACCAGTGAAGGGATTTGGTAAAAGGCTTTCTGGTACAGATGTTGAAAGATTAACAAGTAAGAATGCTACTAAAGAATCACTGACGTCAGTACAGCGAAAAGGTAGAGTGAAGGTATCGAAGGCATTTCAAGATCCCCTGTCGACCCTTCAAAGTAACAGCAATGTTGTAGAGCCTATGAATCATTTCAGGTCTGAAGCTGAAGAAGCTGAAGAAAACTTGTCATGGGAAAAAATAAAGAGGAAAATCGACTTTGATGTGGAGGTAACACCGGAAAAAAAAGTGAAGCAGCAGAAGACCAATGCGGCGTCTACTGATTCATTGGGACAGAAACGGTCAAGATCAGGAAGGGTGCTTGTGTCATCACTAGAGTTTTGGCGTAACCAAATTCCTGTTTATGATATGGATCGGAACCTTATCCAAGTAAAGGATGGTAGTGAGACTAACTCCGCTCCA |
| Nsdbd | GCTGCCGTTAAATCTGGCACCAAAGCTAAACGTGCTCAGCGTCCGGCAAAATATAGCTACGTTGACGAAAACGGCGAAACTAAAACCTGGACTGGCCAAGGCCGTACTCCAGCTGTAATCAAAAAAGCAATGGATGAGCAAGGTAAATCCCTCGACGATTTCCTGATCAAGCAA |
| Nsdbd-CENPC-k-Nsdbd | ATGGCTGCCGTTAAATCTGGCACCAAAGCTAAACGTGCTCAGCGTCCGGCAAAATATAGCTACGTTGACGAAAACGGCGAAACTAAAACCTGGACTGGCCAAGGCCGTACTCCAGCTGTAATCAAAAAAGCAATGGATGAGCAAGGTAAATCCCTCGACGATTTCCTGATCAAGCAACAGAAACGGTCAAGATCAGGAAGGGTGCTTGTGTCATCACTAGAGTTTTGGCGTAACCAAATTCCTGTTTATGATATGGATCGGAACCTTATCCAAGTAAAGGCTGCCGTTAAATCTGGCACCAAAGCTAAACGTGCTCAGCGTCCGGCAAAATATAGCTACGTTGACGAAAACGGCGAAACTAAAACCTGGACTGGCCAAGGCCGTACTCCAGCTGTAATCAAAAAAGCAATGGATGAGCAAGGTAAATCCCTCGACGATTTCCTGATCAAGCAA |
| Nsdbd-CENPC-k | ATGGCTGCCGTTAAATCTGGCACCAAAGCTAAACGTGCTCAGCGTCCGGCAAAATATAGCTACGTTGACGAAAACGGCGAAACTAAAACCTGGACTGGCCAAGGCCGTACTCCAGCTGTAATCAAAAAAGCAATGGATGAGCAAGGTAAATCCCTCGACGATTTCCTGATCAAGCAACAGAAACGGTCAAGATCAGGAAGGGTGCTTGTGTCATCACTAGAGTTTTGGCGTAACCAAATTCCTGTTTATGATATGGATCGGAACCTTATCCAAGTAAAG |

**Supplementary Table 4: List of primers used in this study**

|  | Forward primer | Reverse primer |
| --- | --- | --- |
| **CENPC-k motif constructs** |  |  |
| ATG-CENPC-k-EYFP | GGGGACAAGTTTGTACAAAAAAGCAGGCTTCATGCAGAAACGGTCAAGATCAGG | GGGGACCACTTTGTACAAGAAAGCTGGGTCCTTTACTTGGATAAGGTTCC |
| EYFP-CENPC-k | GGGGACAAGTTTGTACAAAAAAGCAGGCTTCCAGAAACGGTCAAGATCAGG | GGGGACCACTTTGTACAAGAAAGCTGGGTCCTTTACTTGGATAAGGTTCC |
| **CENPC motif constructs** |  |  |
| ATG-CENPC-EYFP | GGGGACAAGTTTGTACAAAAAAGCAGGCTTCATGGGTGGTGTCAGACGAAGTAC | GGGGACCACTTTGTACAAGAAAGCTGGGTCAACAGTAGTCAAACTCTCAT |
| EYFP-CENPC | GGGGACAAGTTTGTACAAAAAAGCAGGCTTCGGTGGTGTCAGACGAAGTAC | GGGGACCACTTTGTACAAGAAAGCTGGGTCAACAGTAGTCAAACTCTCAT |
| **CENPC-k_DNAb motif constructs** |  |  |
| ATG-CENPC-k_DNAb | GGGGACAAGTTTGTACAAAAAAGCAGGCTTCATGAAAGGAAAATCAAAGAAAAGTG | GGGGACCACTTTGTACAAGAAAGCTGGGTCtttgattttcaagtttcttcgctt |
| CENPC-k_DNAb | GGGGACAAGTTTGTACAAAAAAGCAGGCTTCAAAGGAAAATCAAAGAAAAGTGAG | GGGGACCACTTTGTACAAGAAAGCTGGGTCtttgattttcaagtttcttcgctt |
| CENPC-k_DNAbΔ478-537 | GGACAGAAACGGTCAAGATCAGGAA | CTTCTCACTTTTCTTTGATTTTCCT |
| CENPC-k_DNAbΔ573-580 | CCATCTAAAGGAAAAGGATCGGATT | CTTTACTTGGATAAGGTTCCGATCC |
| **CENPC_DNAb motif constructs** |  |  |
| ATG-CENPC_DNAb | GGGGACAAGTTTGTACAAAAAAGCAGGCTTCATGAAGAAGCCGAAGAAAACTCT | GGGGACCACTTTGTACAAGAAAGCTGGGTCACCTTCTCCAGGGGATGCAT |
| CENPC_DNAb | GGGGACAAGTTTGTACAAAAAAGCAGGCTTCAAGAAGCCGAAGAAAACTCT | GGGGACCACTTTGTACAAGAAAGCTGGGTCACCTTCTCCAGGGGATGCAT |
| **αKNL2-C deletion constructs** |  |  |
| αKNL2-CΔDNAb(1) | ACCCTTCAAAGTAACAGCAATGTTG | CGACAGGGGATCTTGAAATGCCTTC |
| αKNL2-CΔDNAb(2) | GGGGACAAGTTTGTACAAAAAAGCAGGCTTCATGACGGAACCAAATCTCGACGAAG | GGGGACCACTTTGTACAAGAAAGCTGGGTCTGGAGCGGAGTTAGTCTCACTACC |
| **αKNL2-C in pF3A** |  |  |
| αKNL2-C | GGTTGCGATCGCATGGATTACAAGGATGACGATGACAAGGCAGCCGGTATGAATTACTCTGGGACGAAAGTCA | GTGTGTTTAAACTTATTTGATTTTCAAGTTTCTTCGC |
| αKNL2-CΔDNAb(1) | ACCCTTCAAAGTAACAGCAATGTTG | CGACAGGGGATCTTGAAATGCCTTC |
| αKNL2-CΔDNAb(2) | GAATTCGAGCTCGGTACCAGTGAAG | TGGAGCGGAGTTAGTCTCACTACCA |
| **Nsdbd constructs** |  |  |
| Nsdbd amplification | GCTGCCGTTAAATCTGGCAC | TTACCCAACTTTGCGTTTCTTTTTCGGTTGCTT |
